# Glucosylceramide regulates depression through activating peroxisome proliferator-activated receptor gamma in dorsal striatum

**DOI:** 10.7150/thno.123178

**Published:** 2026-01-01

**Authors:** Linhong Jiang, Yuman He, Haxiaoyu Liu, Dingwen Zhang, Yanping Dai, Qian Bu, Quanshan Shi, Huaichuan Duan, Ying Zhao, Shu Li, Shuang Han, Yuanyi Zhou, Yue Zhao, Feng Qin, Yaxing Chen, Liang Wang, Hongchun Li, Chunqi Liu, Meng Qin, Weihong Kuang, Ni Zhang, Yinglan Zhao, Xiaobo Cen

**Affiliations:** 1 Mental Health Center and National Chengdu Center for Safety Evaluation of Drugs, State Key Laboratory of Biotherapy and Collaborative Innovation Center for Biotherapy, West China Hospital, Sichuan University; Chengdu, 610041, China.; 2 Key Laboratory of Medicinal and Edible Plants Resources Development of Sichuan Education Department, School of Pharmacy, Chengdu University; Chengdu, 610106, China.; 3 Institute for Breast Health Medicine, State Key Laboratory of Biotherapy, West China Hospital, Sichuan University and Collaborative Innovation Center; Chengdu, 610041, China.; 4 Department of Psychiatry, West China Hospital, Sichuan University; Chengdu, 610041, China.

**Keywords:** depression, glucosylceramide, peroxisome proliferator-activated receptor gamma, dopamine 2 receptor-expressing medium spiny neurons, dorsal striatum

## Abstract

**Rationale:** Depression is a heterogeneous disorder influenced by cell type-specific gene transcription in the brain. Peroxisome proliferator-activated receptor gamma (PPARγ) plays an important role in modulating the pathophysiology of depression. However, the role of PPARγ signaling in modulating depression-responsive neuronal ensembles remains largely unknown.

**Methods:** We established a chronic restraint stress mouse model and integrated multi-omics and functional approaches to investigate the role of glucosylceramide (GlcCer)-PPARγ signaling in stress-induced depression. Conditional knockout mice targeting glucosylceramide synthase (GCS) or *Pparg* in dopamine D2 receptor-expressing medium spiny neurons (D2-MSNs) were generated using a Cre-loxP system, and molecular assays were used to characterize GlcCer as an endogenous activator of PPARγ-driven transcriptional programs.

**Results:** GlcCer as a crucial native activator of PPARγ that specifically modulates depression by binding to the activation function 1 domain of PPARγ in D2-MSNs in the dorsal striatum. Genetic ablation of GCS in D2-MSNs disrupts PPARγ signaling and neuronal function, leading to depression-like behaviors in mice. Selective deletion of *Pparg* in D2-MSNs produces a similar effect through dopamine D2 receptor. Administration of GlcCer or the PPARγ agonist pioglitazone reverses stress-induced depression-like behaviors, combined GlcCer and pioglitazone exerts additive antidepressant effects.

**Conclusions:** These findings demonstrate a pivotal role for GlcCer-PPARγ signaling in D2-MSNs in depression, highlighting the therapeutic potential of targeting PPARγ activity in depression.

## Introduction

Depression is one of the most heterogeneous and disabling neurological conditions worldwide, and stress is a major risk factor for depression 1, 2. Dysregulated transcription factor-mediated gene expression is considered one of the molecular bases of depression 3-5. Among transcription factors, peroxisome proliferator-activated receptor gamma (PPARγ), a ligand-activated nuclear transcription factor, has been shown to be an important player in modulating the pathophysiology of depression. PPARγ is prominently expressed in GABAergic neurons and microglia across mood-regulating brain regions, including the dorsal striatum, nucleus accumbens, and hippocampus 6, 7. PPARγ activation attenuates stress responses and ameliorates depression-like behaviors in animal models 8, 9. Clinically, PPARγ agonists, particularly thiazolidinediones, such as pioglitazone, have proven effective in alleviating symptoms of depression and bipolar disorder 10. These findings indicate that modulating PPARγ activity may be a promising therapeutic strategy for depression 11, 12; however, the role of PPARγ signaling in modulating depression-responsive neuronal ensembles remains largely unknown. In addition, the undesirable side effects of thiazolidinediones, such as cardiovascular failure, liver toxicity, weight gain and decreased urine glucose levels 13, 14, greatly limit their clinical use for treating depression. Thus, activating PPARγ with a native ligand or activator may facilitate optimal propagation of endogenous PPARγ signals without synthetic agonist-induced PPARγ overstimulation and/or off-target effects 15, 16. Furthermore, given the neuronal heterogeneity and complexity of brain regions 17, 18, unraveling how PPARγ signaling pathways function within specific neuronal subtypes is crucial for the precise development of PPARγ-targeted therapies for depression.

Glucosylceramide (GlcCer), the main precursor of complex glycosphingolipids, plays critical roles in the structure of plasma membranes and signaling pathways 19, 20. Here, we demonstrate that GlcCer regulates depression-like behaviors by activating PPARγ independent of PPARγ agonists in dopamine 2 receptor-expressing medium spiny neurons (D2-MSNs) in the dorsal striatum. Reducing the GlcCer level in D2-MSNs in the dorsal striatum through genetic ablation of GlcCer synthase (GCS) abolishes PPARγ activity and causes depression-like behaviors in mice, and selective deletion of *Pparg* in D2-MSNs produces a similar effect. Combined treatment with GlcCer and pioglitazone produces significant synergistic effects. These findings reveal a critical role for GlcCer-PPARγ signaling in D2-MSNs in depression and pave the way for the exploration of PPARγ-targeted therapies for depression.

## Methods

### Experimental animals

All experimental procedures and use of the mice were in accordance with the study protocols approved by the Institutional Animal Care and Use Committee of Sichuan University (permit number: 20250302077). Male mice aged 2-4 months on a C57BL/6J background were used. Adults male C57BL/6J mice (8-12 weeks old, body weight 20 ± 2 g) were bred and housed in clear plastic cages with 12 h light/dark cycle (07:00/19:00 h) at a constant temperature (20-25℃) with food and water *ad libitum*. Before behavioral testing, mice were acclimated to the conditions for 3 days. *Ugcg*^f/f^ mice and *Pparg*^f/f^ mice (Catalog numbers T008258 and T006940, respectively) were purchased from GemParmatech Co.Ltd (Nanjing, China). These mice were crossed with D1-Cre [MMRRC Tg(Drd1a-cre)EY262Gsat] or D2-Cre [MMRRC Tg(Drd2-cre) ER44Gsat] mice to delete *Ugcg* gene or *Pparg* gene from D1-MSNs or D2-MSNs. *Ugcg*^f/f^ mice were genotyped with forward prime (CTTGGCTAGTGGTCAGTCATCTAGC) and reverse primer (GGAAGCACACCAGTAAGGGAAACA). *Pparg*^f/f^ mice were genotyped with forward primer (CTTCCCCTTCCCCAAAATGAGTC) and reverse primer (TCTGTGGCTGGACTACAGGA).

### Cell lines

Neuro2a (N2a) neuroblastoma cell lines were obtained from ATCC and confirmed to be free of mycoplasma contamination throughout the study. N2a cells were cultured in DMEM supplemented with 10% FBS and cultured in a humidified 5 % CO_2_ atmosphere at 37 °C. Upon reaching confluency, the N2a cells were dissociated with a trypsin-EDTA solution and passaged at a 1:3 ratio.

### Drug

The GCS agonist, (-)-L-threo-PDMP hydrochloride (abbreviated as PDMP in the figures, #10005278, Cayman, USA) was dissolved in saline containing 5% ethanol. GlcCer (#131303, Avanti poplar lipids, USA) was dissolved in saline containing 5% Tween 80. Pioglitazone hydrochloride (abbreviated as pioglitazone in the figures, HY-14601, MedChemExpress, USA) was dissolved in saline containing 5% dimethyl sulfoxide. UNC9995 (abbreviated as UNC in the figures, HY-120920, MedChemExpress, USA) was dissolved in saline containing 5% Tween 80. Clozapine-N-oxide (abbreviated as CNO in the figures, HY-17366, MedChemExpress, USA) was dissolved in saline containing 5% dimethyl sulfoxide.

For intracranial injection, mice were surgically implanted with sterilized guide cannulae in dorsal striatum. Intra-dorsal striatal injection of PDMP (100 μM, 1 μL/injection), pioglitazone (100 μM, 1 μL/injection), UNC (10 μM, 1 μL/injection) was performed 30 min before behavioral test. Mice were intraperitoneally injected with CNO (1 mg/kg/d) before behavioral test.

### Behavioral tests

*Chronic restraint stress (CRS).* For the CRS protocol, male C57BL/6J mice (8-10 weeks old) were obtained from Charles River Laboratories (Beijing, China). Mice were individually placed into well-ventilated 50 mL conical restraint tube (suitable for mice weighing 20-25 g) with multiple holes for 4 h daily between 10:00 AM and 2:00 PM to minimize circadian rhythm variations. The restraint stress procedure was conducted for 14 consecutive days. Then, all behavioral test were performed between 10:00 AM and 18:00 PM.

*Chronic social defeated stress (CSDS).* CD-1 aggressors were screened for aggressive behavior before use. Experimental C57BL/6J mice encountered a novel CD-1aggressor for 10 min daily over 10 consecutive days. Mice were housed opposite a perforated Plexiglas barrier between defeat sessions to enable continuous sensory contact with the aggressor. After 10 days, experimental mice were singly housed overnight and underwent social interaction testing the following day. Then, it was tested by behavior test to divide into resistant mice group and susceptible mice group.

*Open field test (OFT).* Mice were placed in an open chamber (40 × 40 × 30 cm) with black plastic walls, allowing free movement. Mice were gently placed in the center and allowed to explore the area for 5 min. The total distance travelled and time spent in the center (20 × 20 cm^2^) were recorded using EthoVision version 7.0 software (Noldus Information Technology, Netherlands).

*Elevated plus maze test (EPMT).* The apparatus consisted of two open arms (30 cm × 5 cm) and two closed arms (30 cm × 5 cm × 15 cm) extending from a central platform (5 cm × 5 cm), elevated 50 cm above the floor. Mice were placed on the central platform facing one of the open arms and allowed to explore the maze for 5 min. Behavior was recorded using a video camera positioned above the maze in conjunction with Anymaze tracking software (Stoelting Co.; Wood Dale, IL, USA).

*Light-dark Transition test (LDT).* The apparatus consisted of a rectangular box (44 cm × 21 cm × 21 cm) divided into two compartments: one brightly illuminated (light box, 30 cm × 21 cm) and the other dark (dark box, 14 cm × 21 cm). The light box was illuminated with a 60-watt light source positioned approximately 30 cm above the apparatus, while the dark box was painted black and enclosed to block external light. Each mouse was initially placed in the center of the light box and allowed to explore the apparatus for 5 min. Behavior was recorded by Anymaze tracking software (Stoelting Co.; Wood Dale, IL, USA).

*Sucrose preference test (SPT).* Mice were acclimated to a two-bottle choice paradigm for 48 hours, during which they had access to two bottles of water. The positions of the bottles were switched every 12 h to prevent side preference. Following the acclimation period, mice were subjected to 24 h of water deprivation, during which they had no access to water or sucrose solution. After the 24 h water deprivation period, mice were given access to two bottles: one containing 1% sucrose solution and the other containing plain water. Mice were allowed to freely choose between the sucrose solution and plain water for a testing period of 16 h. The volume of sucrose solution and water consumed was measured at the end of the test.

*Forced swimming test (FST).* Each mouse was placed individually into a water-filled cylinder maintained at 23-25 °C and allowed to swim for 6 min. The first 2 min were considered an acclimation period, and data from this period were not included in the analysis. During the remaining 4 min, the behavior of the mice was recorded using EthoVision version 7.0 software (Noldus Information Technology, Netherlands).

### 3D motion-capture system and behavior decomposition framework

The experimental setup followed a similar design to a previous study 21. In brief, we employed a 3D-motion learning framework and a self-developed software, Behavior Atlas, which utilized a parallel motion decomposition strategy to quantify defensive behavioral phenotypes. Cameras positioned on all four sides of the apparatus simultaneously recorded the spontaneous behaviors of mice. A machine learning-based approach was applied to automatically identify these behavioral phenotypes. Unsupervised behavioral movement clusters were subsequently refined through supervised classification. Behavioral fractions were calculated as the total duration of all movement types, and behavioral transitions were defined as the shift from one movement type to another.

### Sphingolipidomics analysis

The procedure for the extraction of sphingolipids was established according to the LIPID MAPS protocol with modifications 22. Briefly, dorsal striatal tissues were added 0.5 mL of MeOH, 0.25 mL of CHCl_3_ and 7.5 μL of internal standards cocktail (2.5 μM). For sphingolipidomics, 5 μL of internal standards mixture (LM6002, Avanti polar lipids) was added. The mixture was then ultrasonicated for 45 s at room temperature and incubation at 48°C overnight. To avoid potentially interfering glycerolipids, 75 μL of KOH in MeOH (1M) was added, followed by incubation in a heat block at 200 rpm for 2 h at 37 °C. After neutralization, a four-step extraction process was performed to obtain SPL extract for analysis. The supernatant was transferred to a new Eppendorf tube and the resides were added l mL of MeOH and 2 mL of CHCl_3_, followed by centrifugation at 3800 rpm for 8 min. The upper layer was collected, and the lower layer was dissolved in 0.4 mL of MeOH: CHCl_3_ mixture (*v:v* = 2:1) and added 1 mL of CHCl_3_. After centrifugation at 3800 rpm for 8 min, the lower layer was collected. The upper layer was added 1 mL of CHCl_3_ and centrifuged, and the lower layer was transferred to a tube. The extract was dried under a gentle stream of nitrogen and stored at -80 °C for analysis.

Sphingolipidomics were measured with ultra-high-performance liquid chromatography (UPLC) coupled with hybrid Quadrupole-Orbitrap high resolution mass spectrometry (Q-Exactive-HRMS). A Waters CSH C18 column (100 × 2.1 mm, 1.8 μm) was used to separate the SPLs. The mobile phase A consisted of MeOH/H2O/HCOOH (60:40:0.2, *v/v/v*), The mobile phase B consisted of MeOH/IPA/HCOOH (60:40:0.2, *v/v/v*), both containing 10 mM NH4Ac. A linear gradient was optimized as follows (flow rate, 0.4 mL/min): 0-3 min, 5%-10% B; 3-5 min, 10%-40% B; 5-5.3 min, 40%-55% B; 5.3-8 min, 55%-60% B; 8-8.5 min, 60%-80% B; 8.5-10.5 min, 80%-80% B; 10.5-16 min, 80%-90% B; 16-19 min, 90%-90% B; 19-22 min, 90%-95% B; 22-24 min, 95%-95% B; 24-25 min, 95%-5% B, equilibration with 5% B. The injection volume was 2 μL, and the column temperature at 55 °C.

For GlcCers quantitation, brain tissues were quantified by UPLC-MS/MS using a Q-Exactive-HRMS with UPLC. Calibration curves were built for the GlcCer species (C16:0, C18:0, C24:1) using C12-GlcCer as standard. Quantification of GlcCers with various fatty acid chain lengths were achieved using the calibration curve of each GlcCer species with closest number of chain length. The total GlcCers in the tissues were normalized to 1 mg of protein.

### RNA isolation and quantitative reverse transcription-PCR detection

Dorsal striatal tissues were processed in accordance with the RNA isolation kit's instructions (RC102/RC112; Vazyme Biotech Co.,Ltd), mRNA was reverse transcribed using HiScript® III All-in-one RT SuperMix (Vazyme Biotech Co.,Ltd), and cDNA was amplified using SYBR Green (Vazyme Biotech Co.,Ltd). The 2^-ΔΔCt^ method was used to analyze gene expression, and the relative level of the target gene was normalized to *Gapdh*. The primer sequences can be found in Supplemental materials Table S1.

### RNA-sequencing

Total RNA was extracted from dorsal striatal tissues using TRIzol reagent according to the manufacturer's instructions (Invitrogen, USA). RNA-Seq transcriptome library was prepared by TruSeqTM RNA sample preparation kit from Illumina. Double-stranded cDNA was synthesized with random hexamer primers, subjected to end-repair, phosphorylation and “A” base addition. After quantification, paired-end RNA-seq transcriptome libraries were sequenced. Raw paired-end reads were trimmed and quality controlled by SeqPrep and Sickle. Clean reads were aligned to the reference genome using HISAT2 software. Mapped reads of each sample were assembled by StringTie. Differential expression gene (DEG) analysis was performed using the DESeq2/DEGseq/EdgeR with *p* < 0.05, DEGs with |Log_2_FC| > 0 were defined as significantly different expressed genes. Functional-enrichment analysis including GO and KEGG were performed to identify which DEGs were significantly enriched in GO terms and metabolic pathways at a Bonferroni-corrected *p* value.

### Western blotting analysis

Brain tissues were cut into small pieces and then centrifuged at 13,000 g for 15 min at 4 °C. Supernatants were collected, and proteins concentrations were determined by BCA assay. Samples were denatured at 95 °C for 5 min and subsequently stored at -80 °C for further use. Protein samples were loaded onto 10% SDS-PAGE and transferred on PVDF membranes. After being blocked with 5% nonfat dry milk in Tris-buffered saline with 0.1% Tween-20 for 1.5 h, the PVDF membranes were incubated with corresponding primary antibodies overnight at 4 °C. Membranes were then incubated with HRP-conjugated secondary antibodies for 1 h at room temperature and measured with chemiluminescence imaging system (ChemiScope; Clinx, China).

### Immunostaining

Brains were post-fixed overnight in 4% paraformaldehyde (PFA) and cryoprotected overnight in 30% sucrose in PBS. Brains were sliced on a microtome at 10 μm. Brain slices were permeabilized with 0.2% Triton X-100 in PBS for 5 min and blocked with 3% normal goat serum overnight with primary antibodies. Alexa 488, 555 or 647-conjugated secondary antibodies were used (Invitrogen, USA), and nuclei were stained with DAPI. A confocal microscope TCS-SP8 (Leica Microsystems, Germany) equipped with a 63x/1.40 OIL objective was used for imaging.

### Lipid-protein pull down assay

Dorsal striatal tissues were cut into small pieces and then centrifuged at 13,000 g for 15 min at 4 °C. Supernatants were collected, and protein concentrations were determined using BCA assay. GlcCer-beads were equilibrated by washing them three times with ice-cold PBS. The clarified cell lysate was added to the equilibrated beads, then the mixture was incubated on a rotator at 4 °C for overnight, use approximately 500 µg of total protein per reaction. After incubation, the beads were washed three times with ice-cold PBS. Following the final wash, the beads were resuspended in SDS-PAGE sample buffer and heat at 95°C for 5 min to elute the bound proteins. The eluted sample was used to western blotting analysis.

### Proximity ligation assay (PLA)

N2a cells grown on glass coverslips were transfected with PPARγ or PPARγ mutants using Lipofectamine 3000 (Invitrogen, USA). Cells were fixed 72 h after transfection in 4% PFA at -20 °C and subjected to PLA using the Duolink *In Situ* Red Starter Kit Mouse/Rabbit (Sigma-Aldrich, USA) according to the manufacturer's instructions. Briefly, fixed cells were blocked and incubated with mouse anti-PPARγ and affinity-purified rabbit IgG against GlcCer (RAS-0011, Glycobiotech, Germany) for 30 min at 37 °C. Cells were then washed and incubated with anti-mouse MINUS and anti-rabbit PLUS PLA probes for 1 h at 37 °C, followed by ligation and amplification to generate a Texas Red PLA fluorescence signal. DNA was labelled with 1 μg/mL DAPI in PBS for 5 min at room temperature and the cells were embedded in Prolong Gold Antifade Mountant. Imaging was performed using a confocal microscope TCS-SP8 (Leica Microsystems, Germany) equipped with a 63x/1.40 OIL objective. The number of PLA puncta were counted by FIJI.

### Recombinant proteins purification

To obtain human PPARγ-LBD (residues 223-505), E. coli BL21 (DE3) cells harboring the PPARγ plasmid (pET28a-6 × His vector) were grown in LB medium supplemented with 50 μg/mL kanamycin. Protein expression was induced overnight at 16 °C with 0.3 mM IPTG after OD600 reached 0.8. Cells were lysed in the buffer containing 20 mM Tris-HCl (pH 8.0), 250 mM NaCl. The fusion protein was affinity-purified by Ni-Sepharose beads (GE Healthcare Life Sciences, USA). Protein was further purified by Superdex 200 increase 10/300 GL column (GE Healthcare Life Sciences, USA).

### Surface plasmon resonance

Prepared human PPARγ-LBD and human PPARγ-full length (Active Motif, USA) were immobilized onto a CM5 sensor chip using amine coupling. Briefly, the protein was diluted to 50 μg/mL in immobilization buffer and covalently attached to the CM5 chip via an Amine Coupling Kit (Cytiva, USA), following the manufacturer's instructions. A channel without immobilized protein was used as a blank control. The positive control, pioglitazone, and the test molecule, GlcCer, were both diluted in running buffer (PBS containing 5% (v/v) DMSO, 3 mM EDTA, and 0.05% (v/v) surfactant P20, pH 7.4). Gradient concentrations of GlcCer were injected over both channels at a flow rate of 30 μL/min, with an association time and dissociation time of 85 s each. To evaluate whether pioglitazone enhances the affinity of GlcCer with PPARγ, gradient concentrations of GlcCer and pioglitazone (2.3 μM) were mixed and injected into both channels. Pioglitazone was subsequently injected to verify the activity of the immobilized PPARγ protein. All steps were performed at 25 °C, and data were analyzed using BIAcore T100 Evaluation Software, employing a steady-state affinity analysis model.

### Bio-layer interferometry

The bio-layer interferometry assay was performed at at 30 °C on the Octet RED96 System (ForteBio, USA) with PBS as the running buffer. Purified PPARγ-LBD were biotinylated using the EZ-Link Sulfo-NHS-LC-Biotinylation Kit (Thermo Fisher Scientific, USA) according to the manufacturer's instructions. The biotinylated PPARγ was then immobilized onto SSA Biosensors (ForteBio, USA), pre-equilibrated in the running buffer. The biosensors were incubated with various concentrations of the GlcCer or pioglitazone, followed by dissociation. The data were analyzed and the binding parameters were determined using the software supplied by the manufacturer.

### Molecular docking

The 3D structure of human PPARγ was predicted using AlphaFold based on its primary sequence. AlphaFold models were used as templates for homology modeling with the *MODELER* module. Structural reliability was assessed using Profile-3D and Ramachandran plot analysis. Molecular docking was performed with CB-Dock2, which integrates curvature-based cavity prediction and AutoDock Vina for docking. The GlcCer structure was prepared as an SDF file, and the PPARγ1 protein structure was prepared as a PDB file. Ligand-receptor docking was performed using CB-Dock2 following the standard procedure, and docking results with the lowest Vina score were selected. Predicted docking poses were analyzed using PyMol (v2.6), and the key active-site residues were identified for mutation.

### Molecular dynamics simulations

Four comparative molecular dynamics simulations were conducted using ff14SB force field and Amber 19 package. The simulated systems including multiple mutations of hPPARγ/ DNA, hPPARγ/ DNA/ pioglitazone, hPPARγ/ DNA/ GlcCer and hPPARγ/ DNA/ pioglitazone/ GlcCer, abbreviated as Mut_D, Mut_DP, Mut_DG and Mut_DPG, respectively. Solvent effects were represented by TIP3P water model, with solutes placed in the octahedral box with a 15.0 Å boundary. Energy minimization was performed in two steps for all systems. The first step included solute-constrained optimization with a force constant of 500 kcal·mol⁻¹·Å⁻², involving 5,000 steps of steepest descent and 5,000 steps of conjugate gradient minimization. The second step involved unconstrained optimization with the same conditions. The energy convergence threshold was set to 0.01 kcal·mol⁻¹·Å⁻². The 10 ns MD simulations were conducted in two stages: (1) a 1 ns solute-constrained MD with a force constant of 10 kcal·mol⁻¹·Å⁻², where temperature was gradually increased from 0 to 300 K; (2) a 9 ns unconstrained MD using the SHAKE algorithm to constrain bonds involving non-hydrogen atoms, with a non-bonded interaction cutoff of 10 Å. The integration time step was 2 fs, and snapshots were sampled every 1 ps, resulting in 10,000 conformations for further analysis. The VMD 1.9.3 package was used to monitor structural dynamics.

The average binding free energies of four systems (Mut_D, Mut_DP, Mut_DG, Mut_DPG) were computed by Molecular Mechanics/Poisson Boltzmann (MM/PBSA) method. A total of 20 snapshots were collected from each MD trajectory at 500 ps intervals between 1 to 10 ns. The binding free energy (∆G_bind_) was calculated as:

∆*G*_bind_ = ∆*H* - *T*∆*S* = (∆*E*_VDW_ + ∆*E*_ELE_ +∆*G*_PBELE_ +∆*G*_PBSUR_) - *T*∆*S*

Where ∆*H* represents the total enthalpy change and *T*∆*S* is the product of absolute temperature and conformational entropy change. Specifically, ∆*E*_VDW_ indicates intramolecular _VDW_ energy under vacuum; ∆*E*_ELE_ refers to the electrostatic fraction; ∆*G*_PBELE_ and ∆*G*_PBSUR_ represent the hydrophilic and hydrophobic part of solvation binding free energy, respectively.

### RNA-scope assay

Fluorescent in situ hybridization (FISH) for Drd1 and Drd2, along with the fluorescence of GlcCer or PPARγ, was performed using RNA-scope Fluorescent Multiplex 2.0 assay and RNA-scope Probes for mice (Mm-Drd1a-C3, Mm-Drd2) as per the manufacturer's instructions (Advanced Cell Diagnostics, USA). Briefly, dorsal striatal frozen slices were fixed in 4% PFA for 15 min and hybridized the following probes to RNA transcripts and fluorophores. GlcCer and PPARγ were stained by primary antibody against GlcCer or PPARγ. Images were taken within 2 weeks of staining on a Leica SP8 confocal microscope to determine the intensity of Drd1, Drd2, GlcCer or PPARγ staining for each DAPI-positive nucleus. Average GlcCer and PPARγ intensity was calculated for a total of four animals per group with at least three slices analyzed per animal. Images were captured at 1024 × 1024 pixels.

### Chromatin immunoprecipitation (ChIP)

ChIP was performed on dorsal striatal punches from fresh brains of two mice were pooled. Tissue was minced and double crosslinked with DSG for 20 min and 1% formaldehyde for 10 min followed by adding glycine at room temperature for 10 min. After homogenizing tissue pellets in PBS, 1 mL of lysis buffer was added. Samples were sonicated to generate 200-500 base pairs fragments and centrifuged at 14,000 g at 4 °C. Supernatants were diluted in a dilution buffer and incubated with 2 mg of anti-PPARγ antibody (sc-72738, Santa Cruz, CA), overnight at 4 °C. To monitor the specificity of ChIP assays, samples were also immunoprecipitated with a specific antibody isotype-matched control IgG. Dynabeads Protein G were added to the supernatant and incubated for 2 h at 4 °C. Beads were recovered and washed sequentially with low salt buffer, high salt buffer, LiCl buffer and TE three times. Elution buffer was added to the washed beads, treated with RNase at 37 °C for 2 h and proteinase K at 65 °C overnight. DNA were purified using a wash buffer and kept at -20 °C overnight. Quantitative PCRs were performed using PowerUp™ SYBR™ Green Supermix (Cat. no. A25743, Invitrogen, USA) according to the manufacturer's protocol. Primers used for ChIP analysis by RT-PCR:* Kcnd1* Forward: 5′-CTCACGAGGCTAGGCAGTTC-3′, *Kcnd1* Reverse: 5′-CCTTGATCGGGTGACTTGTT-3′; *Adora2a* Forward: 5′-TGCAGCTTCTGCCTGTATTT-3′, *Adora2a* Reverse: 5′-AGGAGGAAGGGAGAGAGGTT-3′.

### PPRE-luciferase activity assay

N2a cells were seeded into 24-well plates and cultured for 24 hours before transfection. A DNA mixture containing the PPRE-luciferase reporter plasmid (0.5 μg), PPARγ/ PPARγ mutation plasmid (0.5 μg) and an internal control plasmid pRL-SV-40 (25 ng) was transfected using lipofectamine 3000 transfection reagent (L3000001, Invitrogen, USA) according to the manufacturer's recommendations. After transfection, the cells were incubated for an additional 48 h. Luciferase activity of the cell lysates was measured using the Dual-Luciferase® Reporter Assay System (11402ES, Yeason, China) according to the manufacturer's instructions.

### PPARγ transcription activity assay

To assess the direct effect of GlcCer on PPARγ activation, we performed the in vitro PPARγ binding assay to PPRE by using a PPARγ transcription factor assay kit (No. 10006855, Cayman, USA) according to the manufacturer protocol. Nuclear extracts were prepared from N2a cells using a nuclear extraction kit according to the manufacturer protocol. The binding reaction between PPARγ and PPRE was carried out at 4°C for 16 h using 3 μg of nuclear extracts. The binding activity between PPARγ and PPRE was indicated by the change of optical density (OD) at 405 nm, and an enzyme reaction was measured by HRP-conjugated antibody in complex of PPRE/ PPARγ/antibody.

### Co-Immunoprecipitation (co-IP)

Dorsal striatal tissues were cut into small pieces and centrifuged at 13,000 g for 15 min at 4 °C. Supernatants were collected, and protein concentrations were determined using a BCA assay. Magnetic beads were equilibrated by washing them three times with ice-cold PBS. The binding reaction between ADORA2a primary antibody and magnetic beads was carried out at 4 °C overnight using 500 μg of cell lysate. After incubation, the beads were washed three times with ice-cold PBS and resuspended in SDS-PAGE sample buffer. The resuspension was heated at 95 °C for 5 minutes to elute the bound proteins. The eluted samples were used for western blotting analysis.

### Designer receptor exclusively activated by designer drug (DREADD)

D2-Cre or D2-*Ugcg*^f/f^ mice were bilaterally injected with AAV-hM3Dq into the dorsal striatum (AP +0.8 mm, ML ±1.5 mm, DV -3.0 mm). Following surgery, mice were given 3 weeks to express the virus before starting behavioral tests. Prior to each behavioral test, mice received an injection of Clozapine N-oxide (CNO; 1 mg/kg,* i.p.*; Sigma-Aldrich, USA) 30 min before the testing session.

### Fiber-photometry

Following injection of the virus (300 nL AAV2/9-DIO-GCaMp6m-WPRE-hGHpA) into the dorsal striatum (AP +0.8 mm, ML ±1.5 mm, DV -3.0 mm), a fiberoptic implant was advanced and secured in the same location in D2-*Ugcg*^f/f^ or D2-*Pparg*^f/f^ mice. To record fluorescence signals, a 488 nm laser beam from a laser tube was reflected by a dichroic mirror, focused by a 10× (NA = 0.3) lens, and coupled to an optical commutator. Continuous video recording and fiber photometry acquisition were conducted during the EPMT or FST. To minimize photobleaching, the laser power at the fiber tip was adjusted to 30 μW. Bulk fluorescence signals were then acquired and analyzed with MATLAB software.

### Viral injection and spine analysis

Mice were anesthetized with sodium pentobarbital and placed in a stereotaxic apparatus. Under sterile conditions, the skull of each mouse was exposed and bregma was identified. A virus of AAV-DIO-mCherry was used to construct the morphology of *Ugcg* gene knockout D2-MSNs. AAV-DIO-mCherry (200 nL) was delivered at a rate of 20 nL/min infused into the dorsal striatum in D2-Cre or D2-*Ugcg*^f/f^ mice, and the virus was allowed to diffuse for 10 min post-injection before needle extraction. After behavioral training, mice were perfused with PBS followed by 4% PFA. Brains were post-fixed overnight in 4% PFA were cryoprotected overnight in 30% sucrose. Brains were sliced on a microtome at 50 μm, the slices were observed with Leica SP8 confocal microscope, and images were taken with a resolution of 1024 × 1024. An average of two dendrites per neuron on five neurons per mouse (n = 4 mice) were analyzed.

### Statistical analysis

All statistics were performed in GraphPad Prism 8 (GraphPad Software Inc., USA). For experiments that included two groups, the results were analyzed using two-side unpaired t-tests. Multiple groups were analyzed using one-way or two-way analysis of variance (ANOVA) followed by Tukey's multiple or Bonferroni's comparisons test. *p* values < 0.05 was considered to be significant. All data are presented as mean ± SEM.

## Results

### GlcCer production is correlated with depression-like behaviors

**Figure 1:**
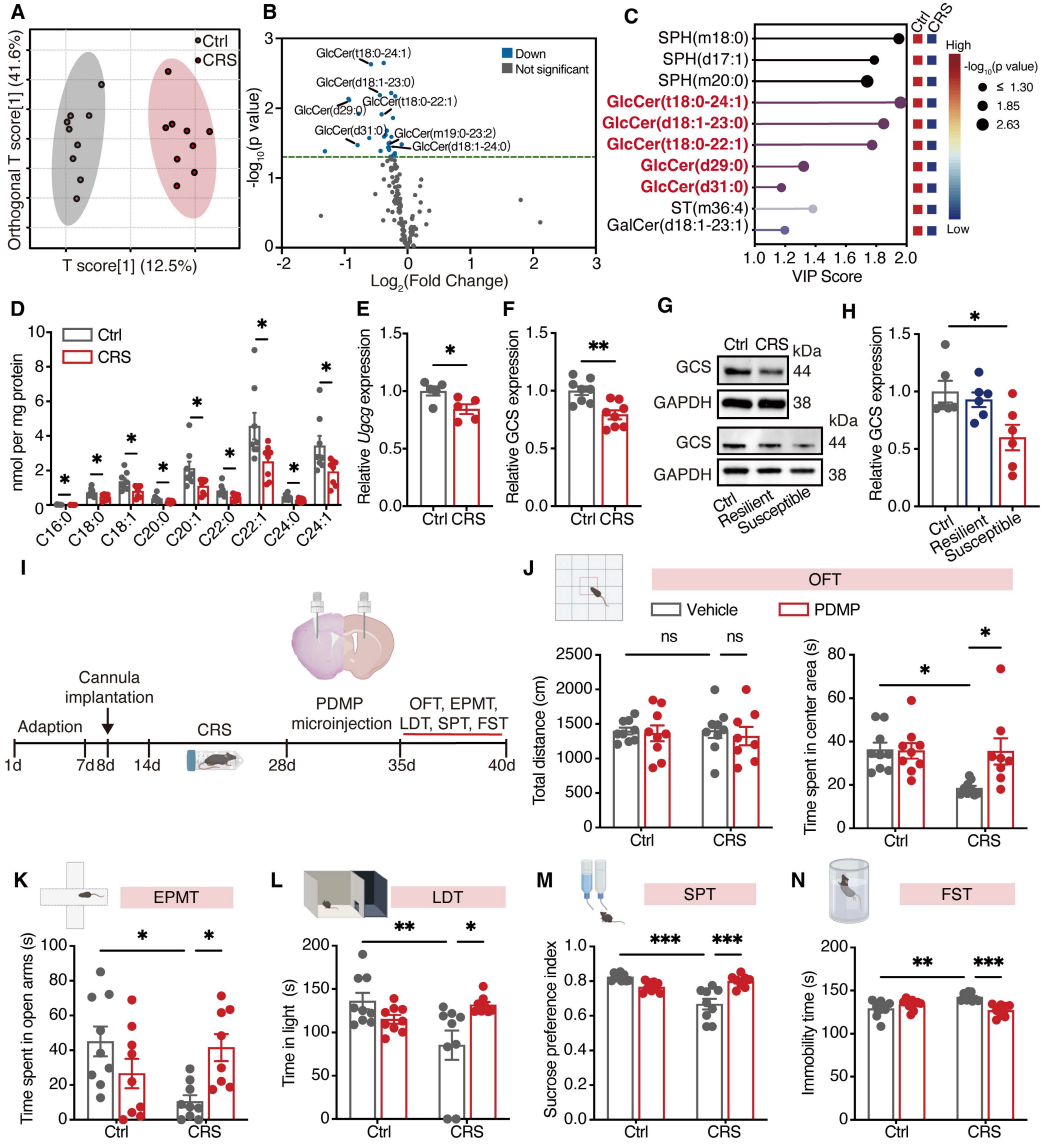
** GCS agonist alleviates CRS-induced anxiety- and depression-like behaviors in the mice. (A)** OPLS-DA illustrates the differential effect of CRS on sphingolipidomic profiles in the dorsal striatum. Gray dots represent samples from the control (Ctrl) group, while red dots represent samples from the CRS group. n = 9. **(B)** Volcano plot visualizes differentially abundant lipids in the dorsal striatum, with significantly downregulated lipids (in blue). **(C)** Variable importance in projection analysis shows the top ten differentially abundant lipids in the dorsal striatum after CRS.** (D)** Targeted UPLC-MS/MS analysis of GlcCer levels with different carbon chain lengths in the dorsal striatum of Ctrl and CRS-induced mice. n = 8. **(E)** qRT-PCR analysis of *Ugcg* (encoding GCS) in the dorsal striatum of Ctrl and CRS-induced mice. n = 5. **(F, G)** GCS protein levels in the dorsal striatum of mice following CRS. n = 8. **(H)** GCS protein levels in the dorsal striatum of mice after CSDS. n = 6. **(I)** Schematic representation of experimental paradigm. PDMP, a GCS agonist, was infused into the dorsal striatum of mice via a cannula.** (J)** The total distance travelled and time spent in the center area during the OFT. **(K)** Time spent in the open arms and number of entries into the open arms during the EPMT. **(L)** Time spent in the light zone and frequency of entries into the lights zone during the LDT. **(M)** Sucrose preference index in the SPT. **(N)** Immobility time during the FST. (J-N) Ctrl + Vehicle, Ctrl + PDMP, and CRS + PDMP groups, n = 9; CRS + Vehicle group, n = 8. All data are presented as the mean ± SEM. Statistical significance was assessed by two-sided unpaired t-test (D-F), one-way ANOVA with Dunnet's (H) or Tukey's multiple comparisons test (J-N). *p < 0.05; **p < 0.01; ***p < 0.001; ns, not significant.

Brain lipid dysregulation has been increasingly recognized as a defining feature of depressive disorders 23. Mice were subjected to chronic restraint stress (CRS), a well-established model of anxiety- and depression-like behaviors, followed by UPLC-MS/MS-based sphingolipidomic profiling to characterize lipid alterations in the dorsal striatum and nucleus accumbens. Multivariate analysis revealed distinct lipid profiles across treatment groups, with GlcCer showing the most pronounced reduction among 28 decreased lipids in the dorsal striatum (Figure 1A-C). In contrast, 12 differentially abundant lipids, including GlcCer and ceramide, were identified in the nucleus accumbens (Figure S1A-C). GlcCer accounted for half of the top-ranking lipids in the dorsal striatum, highlighting its major involvement in CRS-induced sphingolipid remodeling (Figure S1D-F). Further targeted UPLC‒MS/MS analysis revealed significantly decreased levels of GlcCer forms with different carbon chain lengths (Figure 1D). However, no significant changes were observed in the nucleus accumbens, amygdala, or hippocampus (Figure S2A-C). Consistently, the mRNA levels of *Ugcg* (encoding GCS) and GCS protein expression in the dorsal striatum were significantly reduced following 14 days of CRS (Figure 1E-G). The protein level of GCS in the dorsal striatum of susceptible mice was also significantly decreased following chronic social defeat stress (Figure 1G, H). These data suggest that reduced GCS/GlcCer levels may contribute to the pathophysiology of depression.

To validate the role of GlcCer in modulating depression-like behaviors, we microinfused (-)-L-threo-PDMP (PDMP), a GCS agonist, into dorsal striatum via a cannula (Figure 1I). PDMP (100 μM, 1 μL/side) treatment significantly ameliorated anxiety- and depression-like behaviors following CRS, as evidenced by an increased time spent in the center area in the OFT (Figure 1J), open arms in the EPMT (Figure 1K), and light area in the LDT (Figure 1L); restored sucrose preference in the SPT (Figure 1M); and a reduced immobility time in the FST (Figure 1N). Moreover, intra-dorsal striatal administration of PDMP significantly enhanced GCS activity in the dorsal striatum of mice following CRS (Figure S2D). These results demonstrate that GlcCer is able to alleviate depression-like behaviors caused by CRS.

### GlcCer specifically expressed in D2-MSNs regulates depression-like behaviors

**Figure 2:**
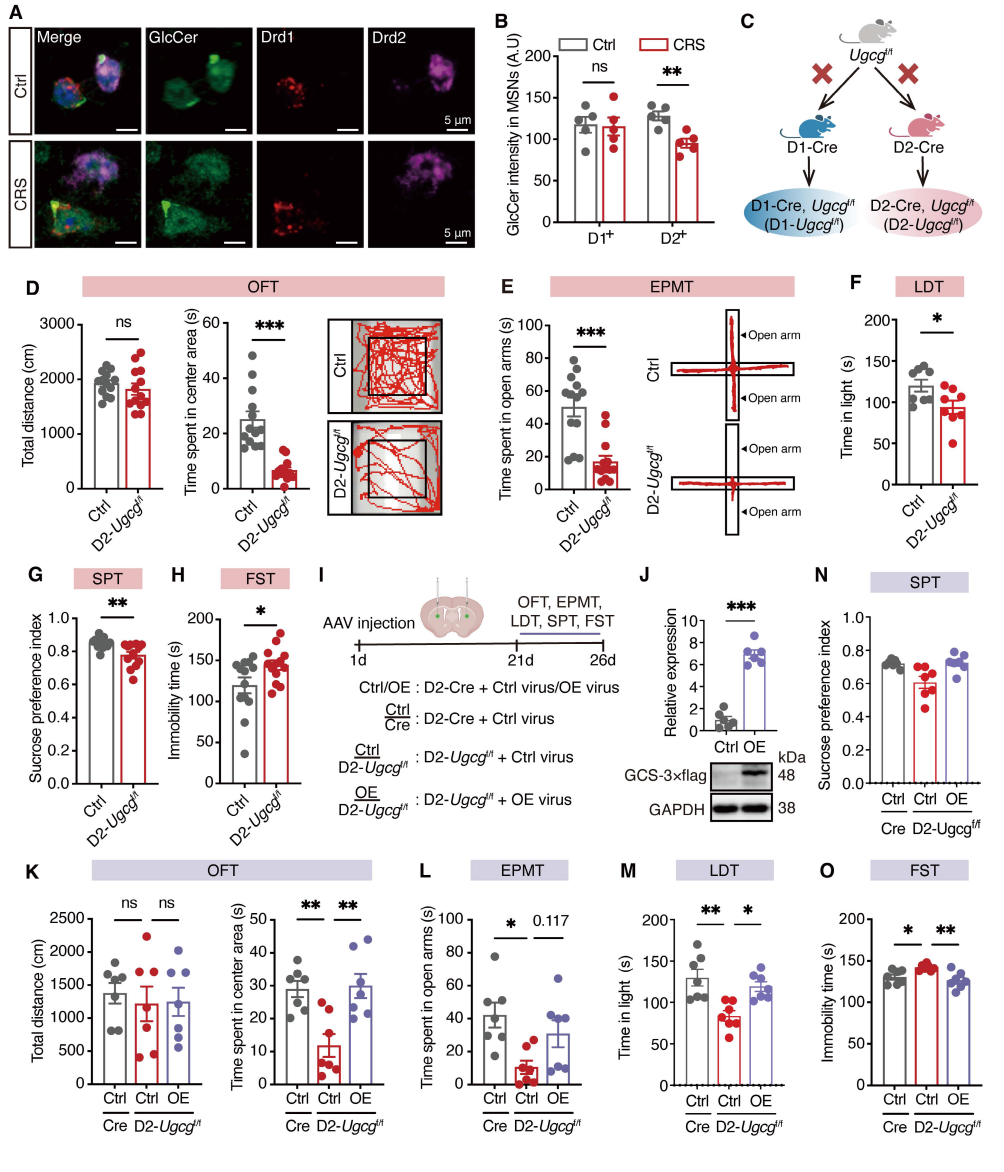
**D2-MSNs-specific loss of *Ugcg* in the dorsal striatum mediates depression-like behaviors. (A, B)** RNA-scope assay shows the representative images (A) and quantification (B) of GCS in D1-positive and D2-positive MSNs in the dorsal striatum of CRS-induced mice. **(C)** Schematic representation of genetic crosses used to delete *Ugcg* in D1-MSNs and D2-MSNs throughout the brain. **(D-H)** Behavioral analysis of D2-*Ugcg*^f/f^ and Ctrl mice in the OFT (D), EPMT (E), LDT (F), SPT (G), FST (H). **(I)** Schematic representation of experimental paradigm. AAV-DIO-eGFP (Ctrl) or AAV-*Ugcg*-DIO-eGFP (OE) was bilaterally microinjected into the dorsal striatum to specifically overexpress *Ugcg* in D2-MSNs. **(J)** Representative images and quantification of GCS expression in the dorsal striatum of D2-Cre mice three weeks after AAV-DIO-*Ugcg* microinjection. n = 6. **(K-O)** Statistical analysis of OE-*Ugcg* and Ctrl mice in OFT (K), EPMT (L), LDT (M), SPT (N), FST (O). n = 7. Data are presented as the mean ± SEM. Statistical significance was assessed by two-sided unpaired t-test (B, D-H and J) or one-way ANOVA with Tukey's multiple comparisons test (K-O). *p < 0.05; **p < 0.01; ***p < 0.001; ns, not significant.

To further investigate the levels of GlcCer in specific neuronal subtypes, we utilized RNA-scope to quantify GlcCer levels in D1-MSNs and D2-MSNs within the dorsal striatum of CRS-induced mice. Interestingly, we observed a significant reduction in GlcCer levels in D2-MSNs but not in D1-MSNs (Figure 2A, B). To further explore the functional consequences of this decrease in GlcCer levels, we generated mice with D1- or D2-specific conditional deletion of the *Ugcg* gene by crossing mice carrying a loxP-flanked *Ugcg* allele (exons 2-5 flanked by loxP sites) with D1-Cre or D2-Cre transgenic mice (Figure 2C, S3A). The immunostaining results confirmed significant reductions in GCS protein levels in both D2-*Ugcg*^f/f^ (Figure S3B, S3C) and D1-*Ugcg*^f/f^ mice (Figure S3D, S3E). Notably, D2-*Ugcg*^f/f^ mice presented increased body weight and food consumption (Figure S3F, S3G), whereas D1-*Ugcg*^f/f^ mice presented no changes in body weight or food consumption (Figure S3H). Therefore, to minimize confounding effects of body weight and age, we selected mice weighing 25 ± 2 g and aged 8-10 weeks for behavioral testing. The behavioral results revealed that D2-*Ugcg*^f/f^ male mice exhibited reduced time spent in the center area during the OFT (Figure 2D), decreased exploration of the open arms in the EPMT (Figure 2E), attenuated time spent in light room during the LDT (Figure 2F), and diminished sucrose preference in the SPT (Figure 2G), along with increased immobility time in the FST (Figure 2H). However, no differences in object exploration in the novel object test (Figure S3I) or the percentage of correct responses in the T-maze test (Figure S3J) were detected when the memory of D2-*Ugcg*^f/f^ mice was assessed. In contrast, female D2-*Ugcg*^f/f^ mice showed a significant reduction in center exploration in the OFT (Figure S4A) but no significant changes in other anxiety- or depression-like behaviors, including those measured by the EPMT, LDT, SPT, or FST (Figure S4B-E). Similarly, male D1-*Ugcg*^f/f^ mice exhibited no significant behavioral alterations (Figure S4F-J), indicating that GlcCer loss in D2-MSNs selectively induced depression-like phenotypes in male mice.

To determine whether restoring* Ugcg* expression in D2-MSNs of the dorsal striatum can reverse depression-like behaviors, we overexpressed *Ugcg* specifically in D2-MSNs of the dorsal striatum via bilateral injection of AAV-DIO-eGFP or AAV-*Ugcg*-DIO-eGFP (Figure 2I). Western blotting analysis confirmed the upregulation of GCS in D2-MSNs of the dorsal striatum after microinjection of AAV-*Ugcg*-DIO-eGFP (Figure 2J). As expected, overexpression of *Ugcg* in D2-*Ugcg*^f/f^ mice reversed the anxiety- and depression-like behaviors in the OFT, EPMT, LDT, SPT and FST (Figure 2K-O). Taken together, these results demonstrate that GlcCer in D2-MSNs specifically regulates depression-like behaviors.

### GlcCer deficiency in D2-MSNs impairs neuronal structural plasticity and reduces calcium activity

**Figure 3:**
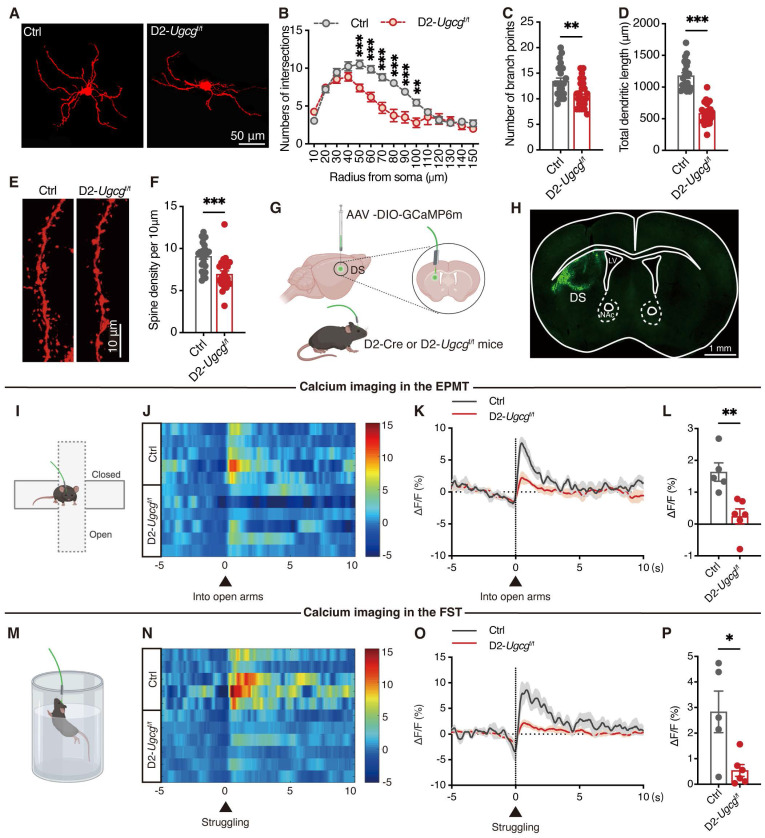
** GlcCer maintains neuronal morphology and calcium activity in D2-MSNs. (A-D)** Representative confocal images of D2-MSNs infected with AAV-DIO-RFP (A), quantification of Sholl analysis (B), number of branch points (C), and total dendritic length (D) in the dorsal striatum of D2-*Ugcg*^f/f^ and Ctrl mice. n = 20 neurons from 4 mice. **(E, F)** Representative images of dendritic segments (E) and quantification of total spine density (F). n = 24 dendrites from 4 mice. **(G)** Schematic of fiber photometry setup.** (H)** Schematic and representative images show fiber placement and GCamp6m expression. **(I)** Schematic of fiber placement for calcium imaging during the EPMT. **(J)** Representative heatmaps of GCamp6m transient ΔF/F events from closed arm to open arm in the EPMT. Each row represents an individual animal. Ctrl: n = 5; D2-*Ugcg*^f/f^: n = 6. **(K, L)** Average (K) and peak (L) ΔF/F changes in the open arms of the EPMT. Ctrl: n = 5; D2-*Ugcg*^f/f^: n = 6. **(M)** Schematic of fiber placement for calcium imaging during the FST. **(N)** Representative heatmaps of GCamp6m transient ΔF/F events during struggling in the FST. Each row represents an individual animal. Ctrl: n = 5; D2-*Ugcg*^f/f^: n = 6. **(O, P)** Average (O) and peak (P) ΔF/F changes during struggling in the FST. Ctrl: n = 5; D2-*Ugcg*^f/f^: n = 6. Data are presented as the mean ± SEM. Statistical significance was assessed using two-sided unpaired t-test (C, D, F, L and P) or two-way ANOVA with Sidak's multiple comparisons test (B). *p < 0.05; **p < 0.01; ***p < 0.001; ns, not significant.

Stress has been shown to reduce calcium signaling in D2-MSNs, ultimately leading to synaptic deficits and neuronal dysfunction 24. To evaluate the impact of GlcCer on dendritic arborization and spine density, we sparingly labeled D2-MSNs in the dorsal striatum of D2-*Ugcg*^f/f^ and control (Ctrl) mice via AAV-DIO-mCherry. Dendritic morphological analysis revealed significant reductions in neuronal branching intersections (Figure 3A, B), the number of dendritic branches (Figure 3C), and dendritic length (Figure 3D) in D2-MSNs of D2-*Ugcg*^f/f^ mice. Additionally, the dendritic spine density of these neurons was significantly decreased (Figure 3E, F). To assess neuronal activity following GlcCer deficiency, we employed *in vivo* fiber photometry combined with the Ca^2+^ indicator GCaMP6m. AAV-DIO-GCaMP6m was injected into the dorsal striatum, and optical fibers were implanted above the infected cells (Figure 3G, H). Fiber photometry revealed that D2-*Ugcg*^f/f^ mice showed reduced Ca²⁺ transients when entering the open arms during the EPMT (Figure 3I-L), and during the struggling phase in the FST (Figure 3M-P).

To explore whether neuronal activity mediates the modulatory effect of GlcCer on depression-like behaviors, we bilaterally microinjected AAV-DIO-hM3D(Gq)-mCherry (AAV-hM3Dq) into the dorsal striatum of D2-*Ugcg*^f/f^ mice to specifically manipulate the activity of D2-MSNs (Figure S5A). Administration of clozapine-N-oxide (CNO, 1 mg/kg, *i.p.*), a ligand for the chemogenetic system, significantly increased the expression of c-Fos, a marker of neuronal activity, in AAV-hM3Dq-injected mice (Figure S5B, C). CNO-treated D2-*Ugcg*^f/f^ mice spent more time in both the center area in the OFT (Figure S5D), the open arms in the EPMT (Figure S5E), the light room in the LDT (Figure S5F) and the index of sucrose preference in the SPT (Figure S5G), but exhibited a lower immobility time in the FST (Figure S5H). These results indicate that GlcCer deficiency induces hypoactivation of Ca²⁺ signaling in D2-MSNs, leading to impaired neuronal function and depression-like behaviors.

### Loss of GlcCer inhibits the PPARγ signaling pathway in D2-MSNs of the dorsal striatum

**Figure 4:**
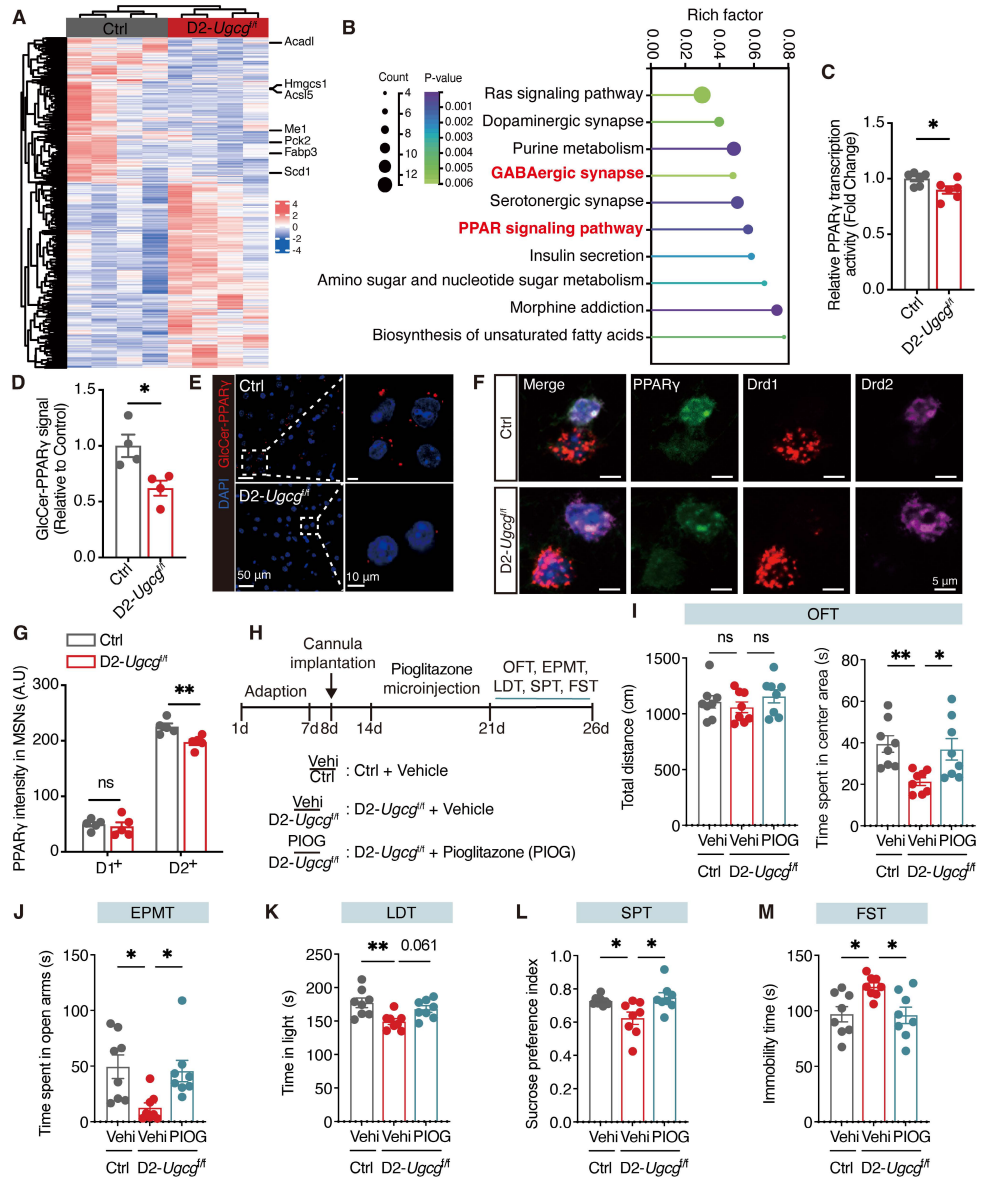
** D2-MSNs GlcCer modulates PPARγ signaling in the dorsal striatum. (A)** Heatmap visualizes the differentially expressed genes (DEGs) in the dorsal striatum between D2-*Ugcg*^f/f^ and Ctrl mice. n = 4.** (B)** KEGG pathway analysis of DEGs. p < 0.05. **(C)** PPARγ transcriptional activity in the dorsal striatum of D2-*Ugcg*^f/f^ and Ctrl mice. n = 6. **(D, E)** PLA analysis shows the quantification (D) and representative images (E) of the GlcCer-PPARγ signal in the dorsal striatum of D2-*Ugcg*^f/f^ and Ctrl mice. n = 4.** (F, G)** RNA-scope assay shows the representative images (F) and quantification (G) of PPARγ in D1-positive and D2-positive MSNs in the dorsal striatum of D2-*Ugcg*^f/f^ and Ctrl mice. n = 5.** (H)** Schematic representation of experimental paradigm. PIOG, a PPARγ agonist, was infused into the dorsal striatum of mice via a cannula. **(I-M)** Statistical analysis of D2-*Ugcg*^f/f^ and Ctrl mice after PIOG microinfusion in OFT (I), EPMT (J), LDT (K), SPT (L), FST (M). n = 8. Data are presented as the mean ± SEM. Statistical significance was assessed using two-sided unpaired t-test (C, D, and G) or one-way ANOVA with Tukey's multiple comparisons test (I-M). *p < 0.05; ns, not significant.

To investigate the impact of the loss of GlcCer on the transcriptional profiles of genes, by RNA sequencing we performed transcriptomic analysis of dorsal striatum tissues from both Ctrl and D2-*Ugcg*^f/f^ mice. We identified 1179 differentially expressed genes (DEGs) between Ctrl and D2-*Ugcg*^f/f^ mice, with 659 genes being upregulated and 520 being downregulated (Figure 4A). KEGG pathway analysis revealed that the top pathways in which the DEGs were enriched included the GABAergic synapse and PPAR signaling pathways (Figure 4B). Further gene set enrichment analysis revealed that the DEGs were significantly enriched in translation at presynapses, postsynapses and synapses (Figure S6A-C). Additionally, the mRNA levels of PPARγ target genes, including *Drd2* and *Kcnd1*, were significantly decreased (Figure S6D). Although both the mRNA and protein levels of PPARγ remained unchanged (Figure S6E-G), its activity was markedly reduced in the dorsal striatum of D2-*Ugcg*^f/f^ mice (Figure 4C). Similarly, PDMP treatment did not alter PPARγ mRNA or protein expression in CRS mice (Figure S6H-J) but significantly enhanced its activity in the dorsal striatum (Figure S6K). These results suggest that GlcCer regulates PPARγ primarily at the level of activity rather than expression. Furthermore, a lipid-protein binding assay further confirmed the decreased interaction between GlcCer and PPARγ in the dorsal striatum of CRS-induced mice (Figure S6L, M). The PLA results revealed a marked reduction in the GlcCer-PPARγ interaction following *Ugcg* deficiency in D2-MSNs of dorsal striatum (Figure 4D, E). RNA-scope analysis indicated that *Ugcg* loss specifically reduced PPARγ levels in D2-MSNs but not in D1-MSNs (Figure 4F, G). Collectively, these findings indicate that the reduction in PPARγ activity in D2-MSNs is a major contributor to depression-like behaviors in D2-*Ugcg*^f/f^ mice.

To determine whether activation of PPARγ in the dorsal striatum could reverse depression-like behaviors in D2-*Ugcg*^f/f^ mice, bilateral cannulas were implanted into the dorsal striatum, followed by microinfusion of pioglitazone (1 μM, 1 μL per side) one week later (Figure 4H). As expected, pioglitazone administration effectively reversed the anxiety- and depression-like behaviors of D2-*Ugcg*^f/f^ mice in the OFT, EPMT, LDT, SPT, and FST (Figure 4I-M). Together, these findings demonstrate that maintaining PPARγ activity in the dorsal striatum is essential for alleviating depression-like behaviors.

### GlcCer activates PPARγ via binding to its activation function 1 (AF1) domain

**Figure 5:**
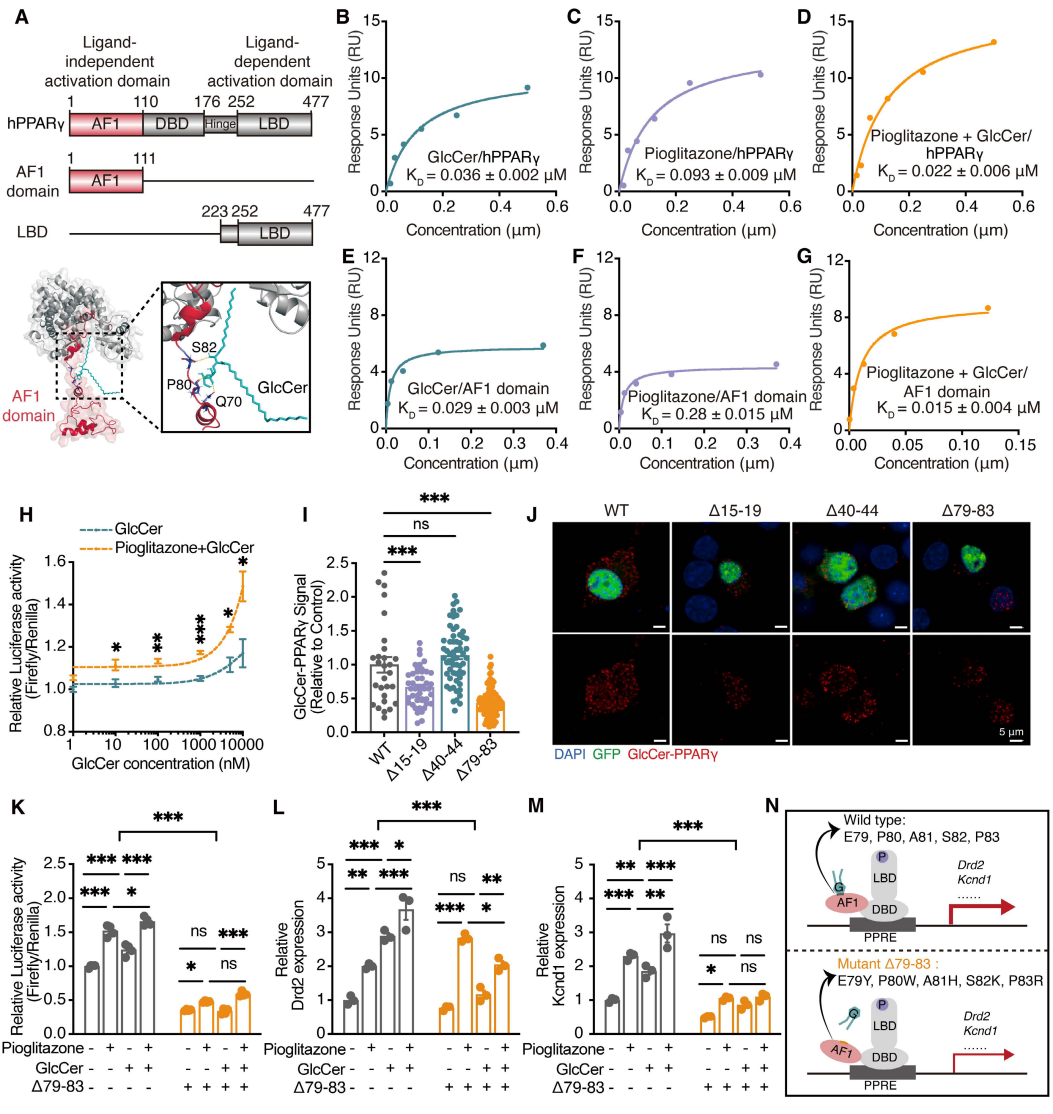
** GlcCer activates PPARγ to regulate the expression of target genes. (A)** Schematic representation of molecular docking between hPPARγ and GlcCer. **(B-D)** SPR was used to determine the equilibrium dissociation constant (K_D_) for the binding affinity of human PPARγ (hPPARγ) to GlcCer (B), pioglitazone (C) and to a mixture of GlcCer and pioglitazone (D). RU, response units. **(E-G)** SPR was used to determine the equilibrium dissociation constant (K_D_) for the binding affinity of human PPARγ AF1 domain to GlcCer (E), pioglitazone (F) and to a mixture of GlcCer and pioglitazone (G). RU, response units. **(H)** PPARγ DNA binding activity in N2a cells treated with GlcCer, pioglitazone or their combination. n = 3. **(I, J)** PLA analysis shows the representative images (I) and quantification (J) of GlcCer-PPARγ signaling in N2a cells after transfected with three mutant plasmids in PPARγ AF1 domain. Wild type, n = 28; Δ15-19, n = 46; Δ40-44, n = 59; Δ79-83, n = 104.** (K)** PPARγ transcriptional activity in N2a cells transfected with *Pparg* Δ79-83, followed by the treatment of GlcCer, pioglitazone or their combination. n = 4.** (L, M)** The mRNA levels of *Drd2* and *Kcnd1* in N2a cells transfected with *Pparg* Δ79-83, followed by the treatment of GlcCer, pioglitazone or their combination. n = 3. **(N)** Schematic of PPARγ transcriptional activity in N2a cells expressing* Pparg* wild type and Δ79-83. Data are presented as the mean ± SEM. Statistical significance was assessed by two-way ANOVA with Tukey's multiple comparisons test (H, K-M), or one-way ANOVA with Tukey's multiple comparisons test (I). *p < 0.05; **p < 0.01; ***p < 0.001; ns, not significant.

To assess whether GlcCer directly binds PPARγ, we measured the binding affinity between human PPARγ (hPPARγ) and GlcCer using surface plasmon resonance (SPR) analysis. GlcCer showed a slightly greater affinity for hPPARγ, with an equilibrium dissociation constant (K_D_) of 0.036 ± 0.002 μM (Figure 5A, B), than did the classical agonist pioglitazone (K_D_ = 0.093 ± 0.009 μM) (Figure 5C). A lipid-protein binding assay further confirmed the direct interaction between PPARγ and GlcCer in the dorsal striatum of mice (Figure S7A). We next performed a SPR assay to evaluate whether pioglitazone enhances the binding affinity of GlcCer for hPPARγ. Different concentrations of GlcCer were pre-incubated with pioglitazone and then flowed over the hPPARγ-immobilized chip surface. Interestingly, the addition of pioglitazone promoted the binding affinity of GlcCer for hPPARγ (K_D_ = 0.022 ± 0.006 μM) (Figure 5D), suggesting that GlcCer and pioglitazone may bind to different regions of PPARγ.

Considering that the AF1 domain and the ligand-binding domain (LBD) of PPARγ are critical functional region 15, we investigated which domain serves as the GlcCer-binding domain. Interestingly, GlcCer showed a stronger affinity for AF1 domain (K_D_ = 0.029 ± 0.003 μM) than pioglitazone (K_D_ = 0.28 ± 0.015 μM) (Figure 5E, F). Furthermore, the addition of pioglitazone enhanced the binding affinity of GlcCer to the AF1 domain of PPARγ (K_D_ = 0.015 ± 0.004 μM) (Figure 5G). Both SPR and biolayer interferometry analyses revealed that GlcCer exhibited minimal binding affinity for the LBD, in contrast with the ligand-dependent agonist pioglitazone, which showed both strong and concentration-dependent binding affinity to the LBD (Figure S7B-E). These findings show that GlcCer specifically binds to the AF1 domain of PPARγ, rather than the classical agonist-binding site on the LBD.

We next assessed the impact of GlcCer on PPARγ transcriptional activity in Neuro-2a (N2a) cells. GlcCer increased PPARγ transcriptional activity in a dose-dependent manner, and this effect was substantially amplified by pioglitazone in N2a cells (Figure 5H). Then, we performed a transcription activity assay with nuclear extracts from N2a cells that were treated with pioglitazone (100 nM), GlcCer (10 μM), or their combination, GlcCer significantly increased PPARγ transcriptional activity, which was further increased upon cotreatment with pioglitazone (Figure S7F).

To further identify the residues that are involved in the GlcCer interaction, we performed AlphaFold molecular modeling (Figure S8A, B) and identified potential hydrogen bonds between GlcCer and residues P80 and Q70, as well as between GlcCer and residues E79 and P82 in the active-site (Figure 5A); additionally, we identified hydrophobic interactions between residues I15 and D19 and between residues F40 and S44 (Figure S8C). Interestingly, almost all of these binding residues are located in the AF1 domain rather than in the LBD, which is consistent with the SPR results. To further study these residues, we generated multiple point mutations in the PPARγ AF1 domain: Δ15-19 (I15K, S16R, S17W, V18K, D19Y), Δ40-44 (F40Y, S41Q, S42R, I43Q, S44F), and Δ79-83 (E79Y, P80W, A81H, S82K, P83R) (Figure S8C). Molecular docking simulations revealed that pioglitazone promoted hydrogen bond formation and reduced binding free energy between DNA and PPARγ Δ79-83, whereas GlcCer had a minimal effect on these interactions (Figure S8D, Table S2, S3). These findings highlight the critical role of the AF1 region in GlcCer-mediated PPARγ-DNA binding.

To further validate these findings, we conducted a proximity ligation assay (PLA), which can detect and visualize protein‒lipid interactions *in situ* (at distances < 40 nm) 25. The results revealed that the GlcCer‒PPARγ interaction was inhibited in N2a cells that were transfected with *Pparg* Δ79-83, which is consistent with the molecular docking predictions results (Figure 5I, J). A dual-luciferase assay confirmed that both pioglitazone and GlcCer increased PPARγ activity in N2a cells that were transfected with wild-type *Pparg*, whereas PPARγ activity was markedly reduced in N2a cells that were transfected with *Pparg* Δ79-83 (Figure 5K). qRT-PCR also revealed a significant reduction in the expression of Dopamine D2 receptor (*Drd2*) and potassium voltage-gated channel subfamily D member 1 (*Kcnd1*), which are two neuron-related target genes of PPARγ, in N2a cells that were transfected with *Pparg* Δ79-83, and this was consistent with the dual-luciferase results (Figure 5L-N). These findings indicate that GlcCer-induced PPARγ transcription dependent on its interaction with the AF1 domain of PPARγ.

To explore whether LBD- and AF1-dependent PPARγ activation differentially regulates the target genes, we measured the mRNA levels of PPARγ target genes in PPARγ-overexpressing N2a cells that were treated with pioglitazone or GlcCer. Our data revealed that both GlcCer and pioglitazone robustly upregulated the expression of adiponectin (*Adipoq*) and *Leptin*, which are key insulin-sensitizing genes that mediate the therapeutic effects of PPARγ activation 26 (Figure S7G). Pioglitazone also strongly induced the expression of adipogenesis-related genes, such as sterol regulatory element-binding protein 1c (*Srebp-1c*), adipose differentiation-related protein (*Adrp*), fatty acid-binding protein 4 (*Fabp4*), fatty acid synthase (*Fasn*) and cluster of differentiation 36 (*Cd36*), which are associated with weight gain 26; however, GlcCer had little or no effect on the expression of these genes (Figure S7G). More interestingly, pioglitazone significantly increased the expression of angiopoietin-like protein 4 (*Angptl4*), which is a key angiogenesis-related gene that is associated with increased cardiovascular risk 27, but GlcCer markedly decreased the expression of *Angptl4* (Figure S7G). These findings show that GlcCer and pioglitazone regulate distinct PPARγ target gene expression by binding to the specific functional domains.

### Targeted GlcCer delivery to the dorsal striatum alleviates depression-like behaviors

**Figure 6:**
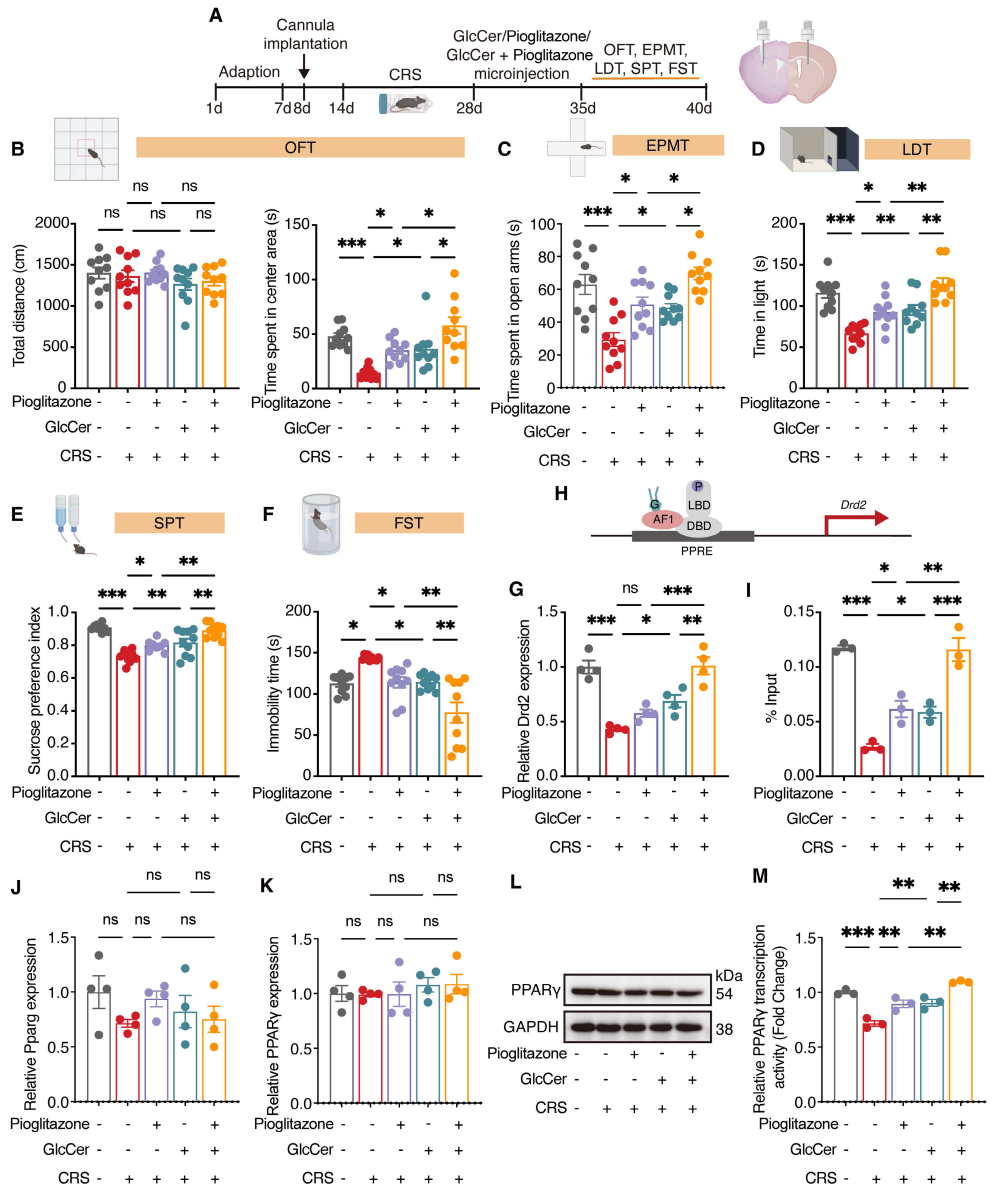
** GlcCer synergistically interacts with PPARγ to mediate depression-like behaviors. (A)** Schematic of the experimental design. GlcCer, pioglitazone or their combination was infused into the dorsal striatum of mice via a cannula. **(B-F)** Behavioral analysis of CRS-induced mice treated with pioglitazone, GlcCer, or their combination in the OFT (B), EPMT (C), LDT (D), SPT (E), and FST (F). n = 10. **(G)** *Drd2* mRNA levels in the dorsal striatum of CRS-induced mice following intra-dorsal striatal infusion of pioglitazone, GlcCer, or their combination. n = 4. **(H, I)** ChIP analysis shows *Drd2* enrichment by PPARγ in the dorsal striatum. n = 3. **(J)** *Pparg* mRNA levels in the dorsal striatum of CRS-induced mice following intra-dorsal striatum infusion of pioglitazone, GlcCer, or their combination. n = 4. **(K, L)** PPARγ protein levels in the dorsal striatum of CRS-induced mice following intra-dorsal striatum infusion of pioglitazone, GlcCer, or their combination. n = 4. **(M)** PPARγ transcriptional activity in the dorsal striatum of CRS-induced mice following intra-dorsal striatum infusion of pioglitazone, GlcCer, or their combination. n = 3. Statistical significance was assessed by one-way ANOVA with Tukey's multiple comparisons test (B-F, G-K, and M). *p < 0.05; **p < 0.01; ***p < 0.001; ns, not significant.

PPARγ is primarily expressed in GABAergic neurons and has been linked to the regulation of mood-related behaviors 6, 8. To examine whether GlcCer supplementation exerts anxiolytic- and antidepressant-like effects through PPARγ activation, cannulas were bilaterally implanted into the dorsal striatum of mice, and one week later, microinfusion of pioglitazone (1 μM, 1 μL/side), GlcCer (200 μM, 1 μL/side), or their combination were administered (Figure 6A). The total distance traveled in the OFT was unaffected by any treatment. However, compared with CRS-induced mice that received vehicle, CRS-induced mice treated with GlcCer exhibited a significant increase in time spent in the center area, with the combination of pioglitazone and GlcCer exhibiting a more pronounced increase (Figure 6B). Similarly, the combined treatment of pioglitazone and GlcCer exhibited significant improvement in behavior in the EPMT (Figure 6C) and LDT (Figure 6D). In assessment of depression-like behavior, CRS-induced mice that received GlcCer presented a marked reduction in anhedonia, as indicated by increased sucrose preference in the SPT, and decreased immobility duration in the FST. These effects were further enhanced by the combination of pioglitazone and GlcCer (Figure 6E, F). In addition, LC-MS analysis revealed that GlcCer levels in the dorsal striatum were increased following treatment with either GlcCer alone or GlcCer combined with pioglitazone, whereas pioglitazone treatment alone did not alter GlcCer levels (Figure S7H). These findings suggest that GlcCer plays an important role in alleviating depression-like behaviors, and its effects is potentiated when combined with pioglitazone treatment.

To confirm the effects of pioglitazone, GlcCer, or their combination on PPARγ transcriptional activity, we quantified the mRNA levels of *Drd2* via qRT‒PCR. Compared with either pioglitazone or GlcCer treatment alone, the combination treatment significantly increased *Drd2* levels in the dorsal striatum of mice after CRS (Figure 6G); chromatin immunoprecipitation (ChIP) also revealed greater enrichment of PPARγ at the* Drd2* promoter induced by combination treatment than treatment of pioglitazone or GlcCer alone (Figure 6H, I). Interestingly, neither pioglitazone, GlcCer, nor their combination altered PPARγ mRNA or protein expression in CRS mice (Figure 6J-L), but all significantly enhanced PPARγ activity in the dorsal striatum (Figure 6M). These results indicate that GlcCer may exert its anxiolytic- and antidepressant-like effects by increasing PPARγ transcriptional activity and that the combination of GlcCer with a PPARγ agonist amplifies these effects.

### PPARγ-DRD2 pathway in D2-MSNs mediates depression-like behaviors

**Figure 7:**
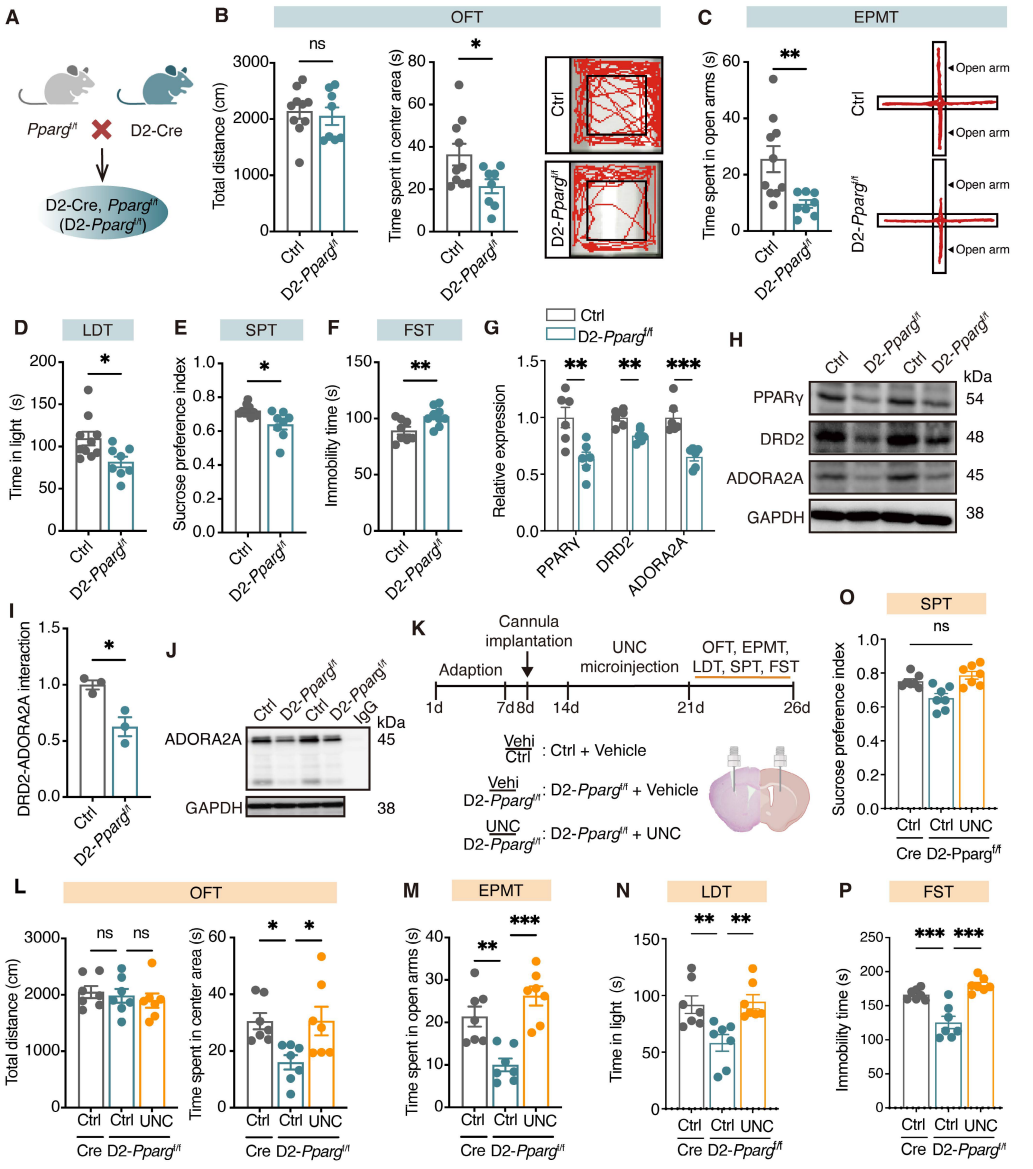
** Loss of *Pparg* in D2-MSNs induces depression-like behaviors through downregulating the expression of DRD2 and ADORA2A. (A)** Schematic representation of genetic crosses used to delete *Pparg* in D2-MSNs throughout the brain.** (B-F)** Behavioral analysis of D2-*Pparg*^f/f^ and Ctrl mice in the OFT (B), EPMT (C), LDT (D), SPT (E), and FST (F). Ctrl mice, n = 10; D2-*Pparg*^f/f^ mice, n = 8. **(G, H)** Expression of PPARγ, DRD2 and ADORA2A in the dorsal striatum of D2-*Pparg*^f/f^ and Ctrl mice. n = 6. **(I, J)** Co-IP analysis shows the interaction between DRD2 and ADORA2A in the dorsal striatum of D2-*Pparg*^f/f^ and Ctrl mice. n = 3. **(K)** Schematic representation of the experimental paradigm. UNC, a DRD2 agonist, was infused into the dorsal striatum of mice via a cannula. **(L-P)** Statistical analysis of vehicle or UNC injection in D2-*Pparg*^f/f^ and Ctrl mice in the OFT (L), EPMT (M), LDT (N), SPT (O), and FST (P). n = 7. Data are presented as the mean ± SEM. Statistical significance was assessed using two-sided unpaired t-test (B-G, and I) or one-way ANOVA with Tukey's multiple comparisons test (L-P). *p < 0.05; ns, not significant.

To determine the role of PPARγ in D2-MSNs in depression, we generated D2-specific conditional *Pparg* knockout mice by crossing mice carrying a loxP-flanked *Pparg* allele (exons 2-3 flanked by loxP sites) with D2-Cre transgenic mice (Figure 7A, Figure S9A). In behavioral tests, male D2-*Pparg*^f/f^ mice spent less time in the center area in the OFT (Figure 7B) and the open arms in the EPMT (Figure 7C) and made fewer entries into and spent less time in the light area in the LDT (Figure 7D). Additionally, these mice presented a reduced sucrose preference in the SPT (Figure 7E) and increased immobility time in the FST (Figure 7F), indicative of pronounced anxiety- and depression-like behaviors. However, female D2-*Pparg*^f/f^ mice (Figure S9B-G) did not show significant changes in anxiety- or depression-like behaviors. Together, these findings demonstrate that the loss of PPARγ in D2-MSNs leads to depression-like phenotypes, underscoring the critical role of PPARγ in maintaining emotional homeostasis.

To assess the naturalistic behaviors of mice following loss of *Pparg* in D2-MSNs, we employed a hierarchical 3D-motion learning framework to analyze the behavioral phenotypes of both Ctrl and D2-*Pparg*^f/f^ mice. The mice were recorded for 15 min to capture spontaneous behaviors, which were subsequently analyzed via a machine learning-based behavior analysis framework. This framework extracted 40 behavioral motifs through unsupervised clustering (Figure S9H, I). By manually grouping these motifs, we identified 14 major behavior types across 18 mice (Figure S9J). Compared with Ctrl mice, D2-*Pparg*^f/f^ mice presented increased grooming (Figure S9K, L) and sniffing *in situ* (Figure S9M) but decreased stretching to climb (Figure S9N), rearing, running, trotting, right turning, walking, and walking with the head up (Figure S9O, P). These findings suggest that loss of *Pparg* in D2-MSNs may induce a state of hypervigilance. Moreover, fiber photometry showed that *Pparg*-deficient in D2-MSNs exhibited decreased Ca²⁺ transients during the struggling phase of the FST (Figure S10A-D).

To further explore whether PPARγ in D2-MSNs modulates depression via its target gene *Drd2*, we measured the protein levels of DRD2 and adenosine A2a receptor (ADORA2A) in the dorsal striatum of D2-*Pparg*^f/f^ mice. Compared with Ctrl mice, D2-*Pparg*^f/f^ mice presented significantly lower levels of DRD2 and ADORA2A in the dorsal striatum (Figure 7G-H). Co-immunoprecipitation analysis confirmed the direct binding of DRD2 with ADORA2A, and *Pparg* deletion in D2-MSNs reduced DRD2 levels, leading to decreased ADORA2A pulldown (Figure 7I, J). Finally, bilateral cannulae were implanted into the dorsal striatum of D2-*Pparg*^f/f^ mice, followed by behavioral testing (Figure 7K). The microinfusion of UNC (10 μM, 1 μL/side), a DRD2 agonist, effectively reversed the reductions of D2-*Pparg*^f/f^ mice in the OFT (Figure 7L), EPMT (Figure 7M), LDT (Figure 7N), SPT (Figure 7O), and FST (Figure 7P), highlighting the critical role of DRD2 in PPARγ-mediated depression-like behaviors. As expected, microinfusion of GlcCer failed to alleviate depression-like behaviors in D2-*Pparg*^f/f^ mice (Figure S10E-J), further supporting the requirement of functional PPARγ signaling for GlcCer-induced antidepressant effects. Collectively, these findings underscore the pivotal role of the PPARγ-DRD2 pathway in D2-MSNs in modulating depression-like behaviors.

## Discussion

PPARγ is recognized as a pivotal yet complex transcription factor, and in addition to exerting metabolic effects in diabetes, PPARγ agonists have shown potential in the treatment of neuropsychiatric disorders 28-31. In this study, we demonstrate that endogenous metabolite GlcCer robustly activates PPARγ through binding the AF1 domain, exerting antidepressant-like effect. Notably, GlcCer-PPARγ signaling is critical for maintaining normal dendritic morphology and neuronal function in D2-MSNs in the dorsal striatum. Furthermore, combined GlcCer and pioglitazone treatment significantly enhanced PPARγ activity, underscoring the substantial antidepressant benefits of this therapeutic strategy.

Several endogenous metabolites, such as polyunsaturated fatty acids, eicosanoids, and 15-deoxy-Δ12,14-prostaglandin J2, have been shown to activate PPARγ and exert anti-neuroinflammatory and antinociceptive effects 32-34. Oral administration of GlcCer has also been shown to alleviate neuroinflammation and memory deficits in aged mice 35, suggesting that GlcCer-mediated signaling may be relevant in these pathophysiological settings. Our data show that GlcCer, which enhances endogenous PPARγ-DRD2 signaling, is a promising therapeutic strategy for treating depression. A unique feature of GlcCer is its specificity in regulating target gene expression, which are different from those of pioglitazone. GlcCer effectively induced the expression of insulin-sensitizing genes, such as *Adipoq* and *Leptin*, to levels comparable to those induced by pioglitazone. Furthermore, GlcCer had no effect on the induction of adipogenic genes (*Fabp4* and *Adrp*) or angiogenesis-related gene (*Angptl4*), which are closely associated with weight gain and increased cardiovascular risk 26, 27. These findings indicate that, compared with traditional LBD-dependent agonists, GlcCer has antidiabetic potential and may possibly mitigate the risk of weight gain and cardiovascular disorders. We consider that the unique interaction of GlcCer with the PPARγ AF1 domain underlies these effects. Further structural studies are needed to elucidate the precise mechanism by which GlcCer targets the AF1 domain of PPARγ.

Interestingly, GlcCer levels remained unchanged in the nucleus accumbens, amygdala, and hippocampus, suggesting that the GlcCer-PPARγ pathway acts in a region-specific manner. Hippocampal PPARγ protects against chronic stress-induced neuroinflammation by maintaining neuronal plasticity and emotional stability 36. Its transcriptional activity is dynamically regulated by co-regulators such as TIP60, which fine-tune PPARγ-dependent gene programs during stress adaptation 9. Thus, hippocampal PPARγ may function through context-dependent modulation rather than sustained expression changes. Consistently, our findings indicate that the GlcCer-PPARγ axis is preferentially engaged in dorsal striatal circuits, while other limbic regions likely employ distinct lipid-receptor mechanisms to preserve emotional homeostasis.

A gap in our understanding remains regarding how GlcCer-PPARγ signaling in D2-MSNs regulates depression-like behaviors. At the molecular level, our data revealed that DRD2 and ADORA2A are key downstream mediators of PPARγ. Given that decreased DRD2-ADORA2A signaling is associated with hypervigilance 37, 38, our findings suggest that reduced DRD2 levels may partly underlie this effect, whereas pharmacologically enhancing DRD2 signaling may have anxiolytic potential. Determining whether DRD2 is necessary component of PPARγ-related behavioral dysregulation requires further investigation. At the neuronal level, D2-MSNs in the dorsal striatum are involved in regulating stress induced anxiety-like behaviors 24. We observed that the loss of GCS impaired synaptic structural plasticity and aberrantly decreased calcium activity in D2-MSNs in the dorsal striatum. Furthermore, selective activation of D2-MSNs in D2-*Ugcg*^f/f^ mice attenuated depressive symptoms. These findings provide compelling evidence connecting GlcCer in the dorsal striatum, GABAergic outputs by D2-MSNs, and depression-like behaviors.

The insights gleaned from our research highlight the therapeutic potential of PPARγ in the brain as a therapeutic target for the treatment of depression, particularly those impacting specific neuronal subtypes. More broadly, our data suggest that GlcCer-PPARγ signaling plays an important role in regulating behaviorally consequential biological functions in the brain. Establishing PPARγ AF1-dependent activation as a novel approach could open new avenues for the development of novel and more targeted therapeutics for depression.

## Conclusions

In summary, we identified GlcCer as an endogenous activator of PPARγ that supports dendritic integrity and neuronal function in D2-MSNs of the dorsal striatum. GlcCer-PPARγ signaling exerts antidepressant-like effects, which are further enhanced by pioglitazone co-treatment, highlighting a promising therapeutic strategy for depression by targeting GlcCer-PPARγ signaling.

## Supplementary Material

Supplementary figures.

## Abbreviations

PPARγ: Peroxisome proliferator-activated receptor gamma
GlcCer: glucosylceramide
GCS: glucosylceramide synthase
D2-MSNs: dopamine D2 receptor-expressing medium spiny neurons
AF1: activation function 1
hPPARγ: human PPARγ
SPR: surface plasmon resonance
LBD: ligand-binding domain
BLI: biolayer interferometry
Drd2: dopamine D2 receptor
Kcnd1: potassium voltage-gated channel subfamily D member 1
Adipoq: adiponectin
Srebp-1c: sterol regulatory element-binding protein 1c
Adrp: adipose differentiation-related protein
Fabp4: fatty acid-binding protein 4
Fasn: fatty acid synthase
Cd36: cluster of differentiation 36
Angptl4: angiopoietin-like protein 4
CRS: chronic restraint stress
OFT: open field test
LDT: light‒dark test
EPMT: elevated plus maze test
SPT: sucrose preference test
FST: forced swimming test
ChIP: chromatin immunoprecipitation
CNO: clozapine-N-oxide
DEGs: differentially expressed genes
ADORA2A: adenosine A2a receptor
