# Supplementary material for: Glucosylceramide regulates depression through activating peroxisome proliferator-activated receptor gamma in dorsal striatum
Source: Theranostics. 2026 Jan 1;16(6):2845–65. doi: 10.7150/thno.123178 (PMC12775823; doi:10.7150/thno.123178)
Supplement: Supplementary file 1 — Supplementary figures. [file thnov16p2845s1.pdf]

Supplementary Information

Glucosylceramide regulates depression through activating peroxisome proliferator-activated receptor gamma in dorsal striatum

Linhong Jiang[1]†, Yuman He[1]†, Haxiaoyu Liu[1], Dingwen Zhang[1], Yanping Dai[1], Qian Bu[1], Quanshan Shi[2], Huaichuan Duan[3], Ying Zhao[1], Shu Li[1], Shuang Han[1], Yuanyi Zhou[1], Yue Zhao[1], Feng Qin[1], Yaxing Chen[1], Liang Wang[1], Hongchun Li[1], Chunqi Liu[1], Meng Qin[1], Weihong Kuang[4], Ni Zhang[4], Yinglan Zhao[1], Xiaobo Cen[1]*

*Corresponding author. Email: xbcen@scu.edu.cn

This PDF file includes:

Figures. S1 to S10

Tables S1 to S3

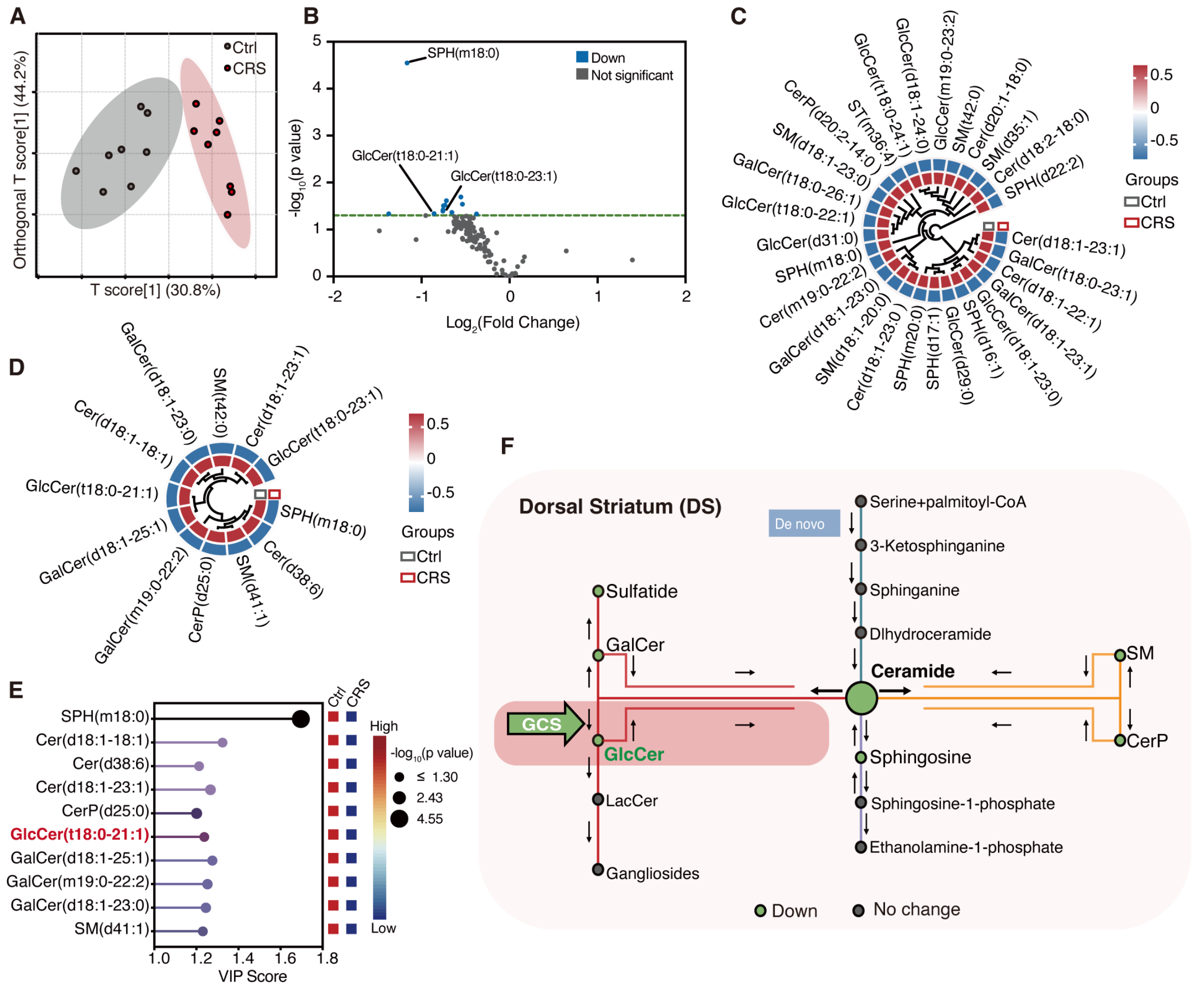

Figure S1. CRS remodels sphingolipid metabolism in the dorsal striatum and NAc. (**A**) OPLS-DA illustrates the differential effect of CRS on sphingolipidomic profiles in the NAc. Gray dots represent samples from the Ctrl group, while red dots represent samples from the CRS group. n = 8. (**B**) Volcano plot visualizes differentially abundant lipids in the NAc, with significantly downregulated lipids (in blue). (**C**) Heatmap shows the differentially abundant lipids in the NAc of mice after CRS. (**D**) Heatmap shows the differentially abundant lipids in the dorsal striatum after CRS. (**E**) Variable importance in projection (VIP) analysis shows the top ten differentially abundant lipids in the dorsal striatum after CRS. (**F**) Changes in sphingolipid metabolites in the dorsal striatum after CRS, with significantly downregulated lipids (in green) and unchanged lipids (in gray).

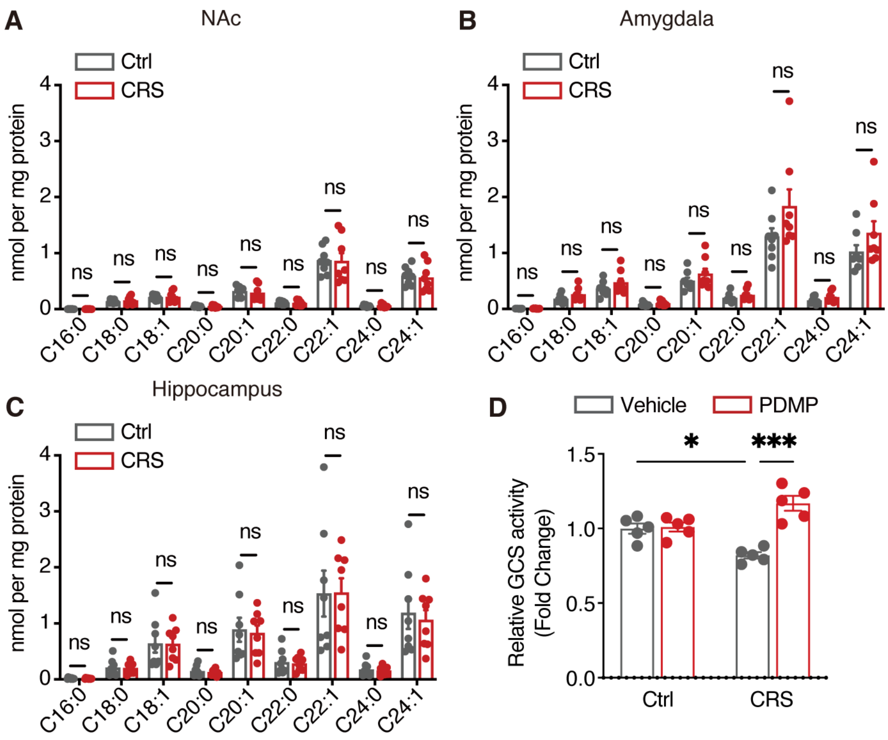

Figure S2. The level of GlcCer in the NAc, amygdala and hippocampus after CRS. (**A-C**) Targeted UPLC-MS/MS was used to examine the level of GlcCer with different carbon chain lengths in the NAc (A), amygdala (B) and hippocampus (C) after CRS. n = 8. **(D)** The GCS activity in the dorsal striatum of CRS-induced mice following intra-dorsal striatum infusion of PDMP. n = 5. Statistical significance was determined using two-sided unpaired t-test (A-C) or one-way ANOVA with Tukey's multiple comparisons test (D). **p < 0.01; ns, not significant.

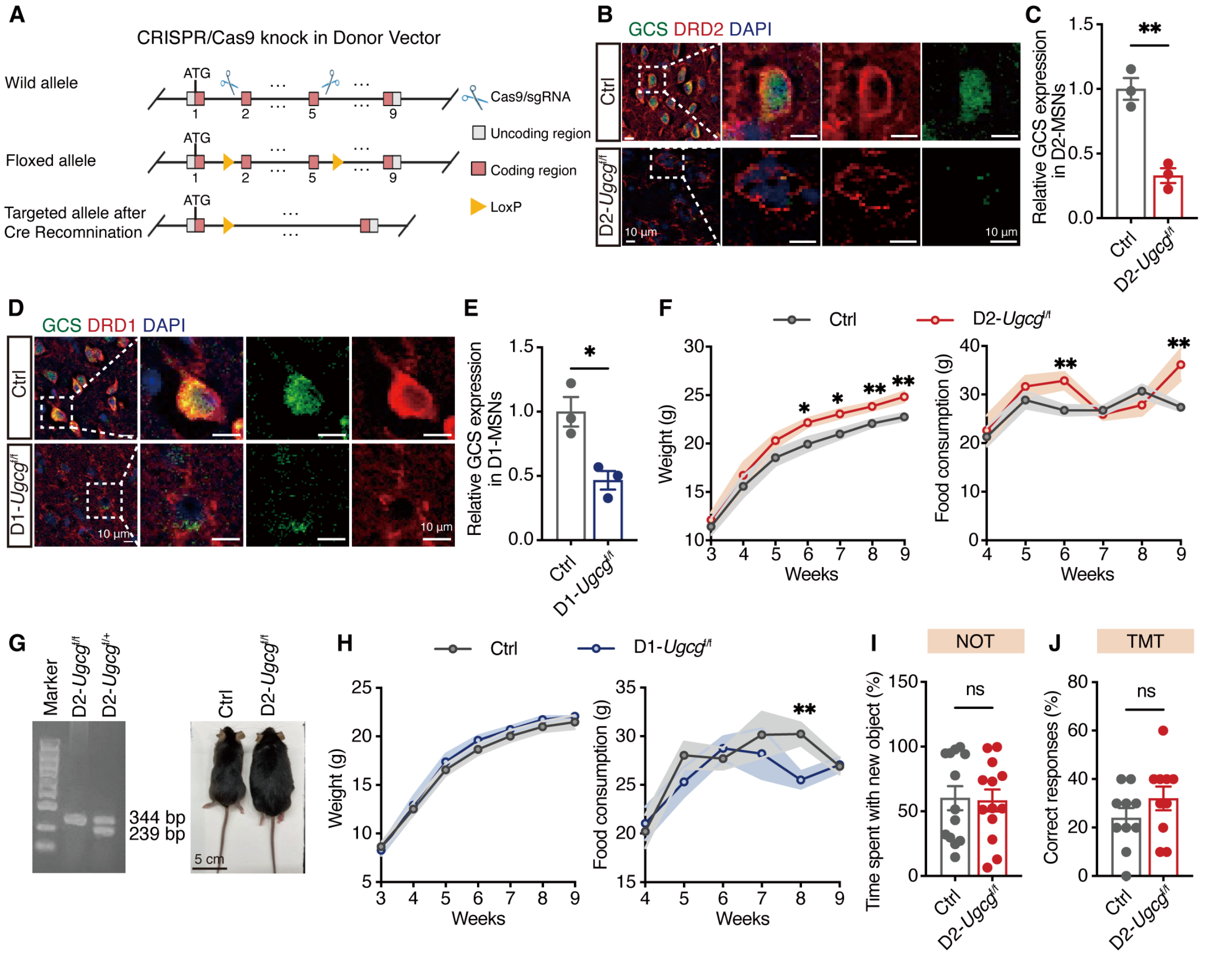

Figure S3. Generation of D1-*Ugcg*$^{f/f}$ and D2-*Ugcg*$^{f/f}$ mice. (**A**) Schematic representation of *Ugcg*$^{f/f}$ gene targeting strategy. (**B**, **C**) Representative images (B) and quantification of the GCS in the D2-positive MSNs (C) from D2-*Ugcg*$^{f/f}$ and Ctrl mice. n = 3. (**D**, **E**) Representative images (D) and quantification of the GCS in the D1-positive MSNs (E) from D1-*Ugcg*$^{f/f}$ and Ctrl mice. n = 3. (**F**) Body weight and food consumption of D2-*Ugcg*$^{f/f}$ and Ctrl mice. Ctrl mice, n = 12; D2-*Ugcg*$^{f/f}$ mice, n = 9. (**G**) Representative PCR genotyping images and appearance of D2-MSNs-specific *Ugcg* homozygous knockout (D2-*Ugcg*$^{f/f}$) and heterozygous knockout (D2-*Ugcg*$^{f/+}$) mice. (**H**) Body weight and food consumption of D1-*Ugcg*$^{f/f}$ and Ctrl mice. Ctrl mice, n = 18; D1-*Ugcg*$^{f/f}$ mice, n = 14. (**I**) Time spent with new objects in the novel object recognition test. n = 13. (**J**) Percentage of corrected responses in the T-maze test. n = 10. Statistical significance was determined using two-sided unpaired t-test (C, E, F and H-J). **p* < 0.05; ***p* < 0.01; ****p* < 0.001; ns, not significant.

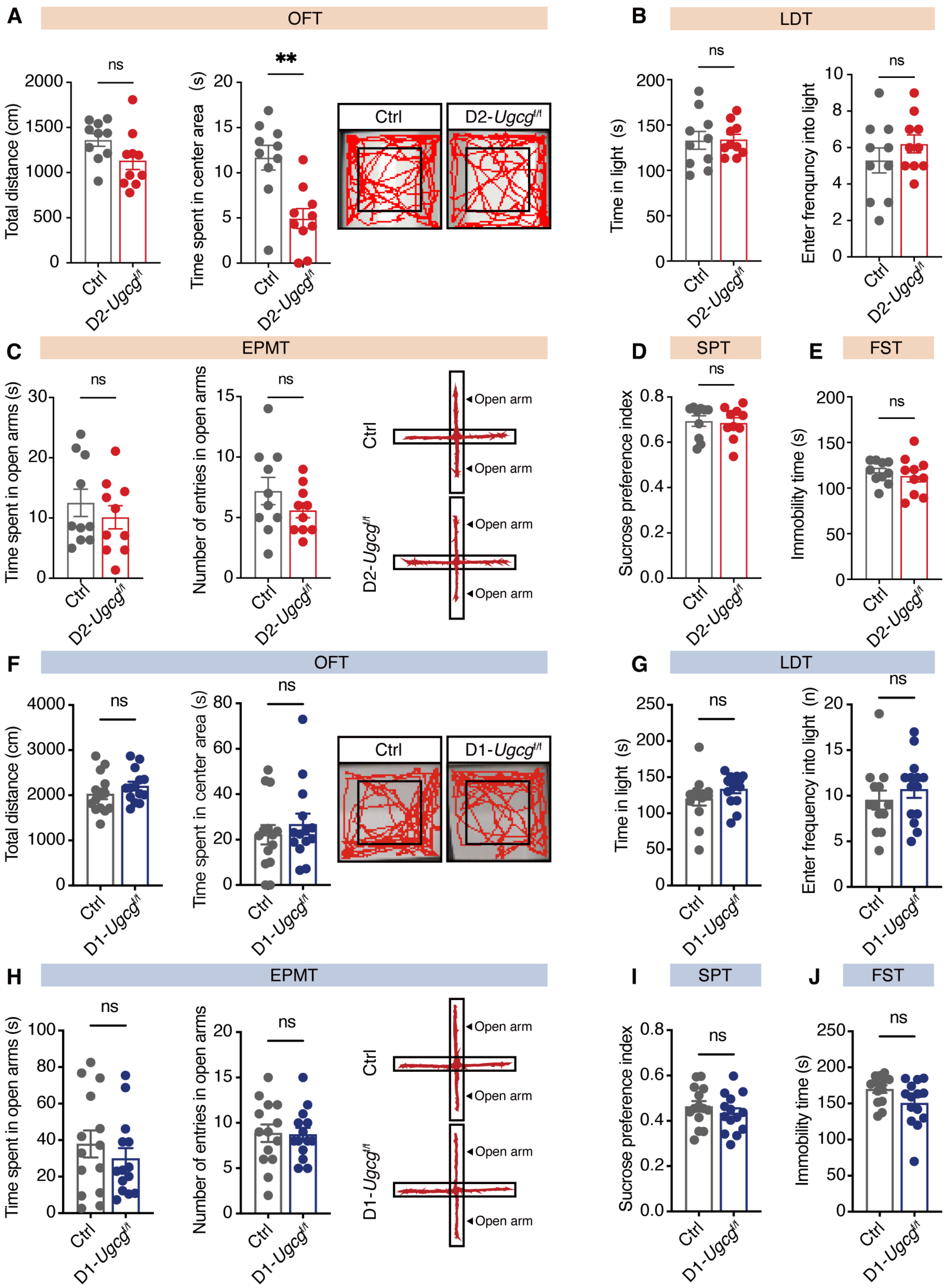

Figure S4. Neurobehavioral assessment of D2-*Ugcg*$^{f/f}$ female mice and D1-*Ugcg*$^{f/f}$ male mice. (**A-E**) Statistical analysis of the Ctrl and D2-*Ugcg*$^{f/f}$ female mice in the OFT (A), LDT (B), EPMT (C), SPT (D), and FST (E). n = 10. (**F-J**) Statistical analysis of the Ctrl and D1-*Ugcg*$^{f/f}$ mice in the OFT (F), LDT (G), EPMT (H), SPT (I), and FST (J). For OFT, EPMT, SPT,

FST, n = 14 mice; for LDT, Ctrl mice, n = 13; D1-*Ugcg*$^{f/f}$ mice, n = 14. Statistical significance was determined using two-sided unpaired t-test (A-J). ns, not significant.

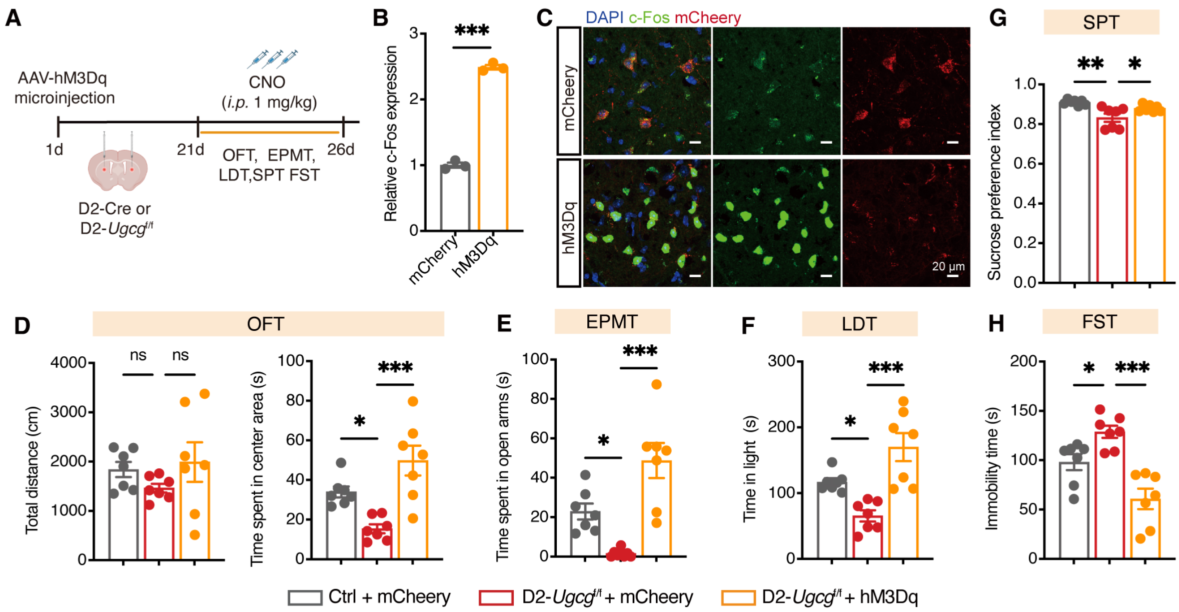

Figure S5. D2-MSNs-specific activation in the dorsal striatum improves depression-like behaviors. (**A**) Schematic representation of experimental paradigm. AAV-DIO-hM3D(Gq)-mCherry (hM3Dq) was bilaterally microinjected into the dorsal striatum of D2-Cre or D2-*Ugcg*$^{f/f}$ mice to specifically manipulate D2-MSNs activity using clozapine-N-oxide (CNO). (**B**, **C**) Representative images (B) and quantification of c-Fos expression (C) in the dorsal striatum of D2-Cre mice. n = 3. (**D-H**) Statistical analysis of D2-*Ugcg*$^{f/f}$ mice injected with AAV-hM3Dq in the OFT (D), EPMT(E), LDT (F), SPT (G), and FST (H). n = 7. All data are presented as the mean ± SEM. Statistical significance was determined using a two-sided unpaired t-test (C) or one-way ANOVA with Tukey's multiple comparisons test (D-H). *p < 0.05; **p < 0.01; ***p < 0.001; ns, not significant.

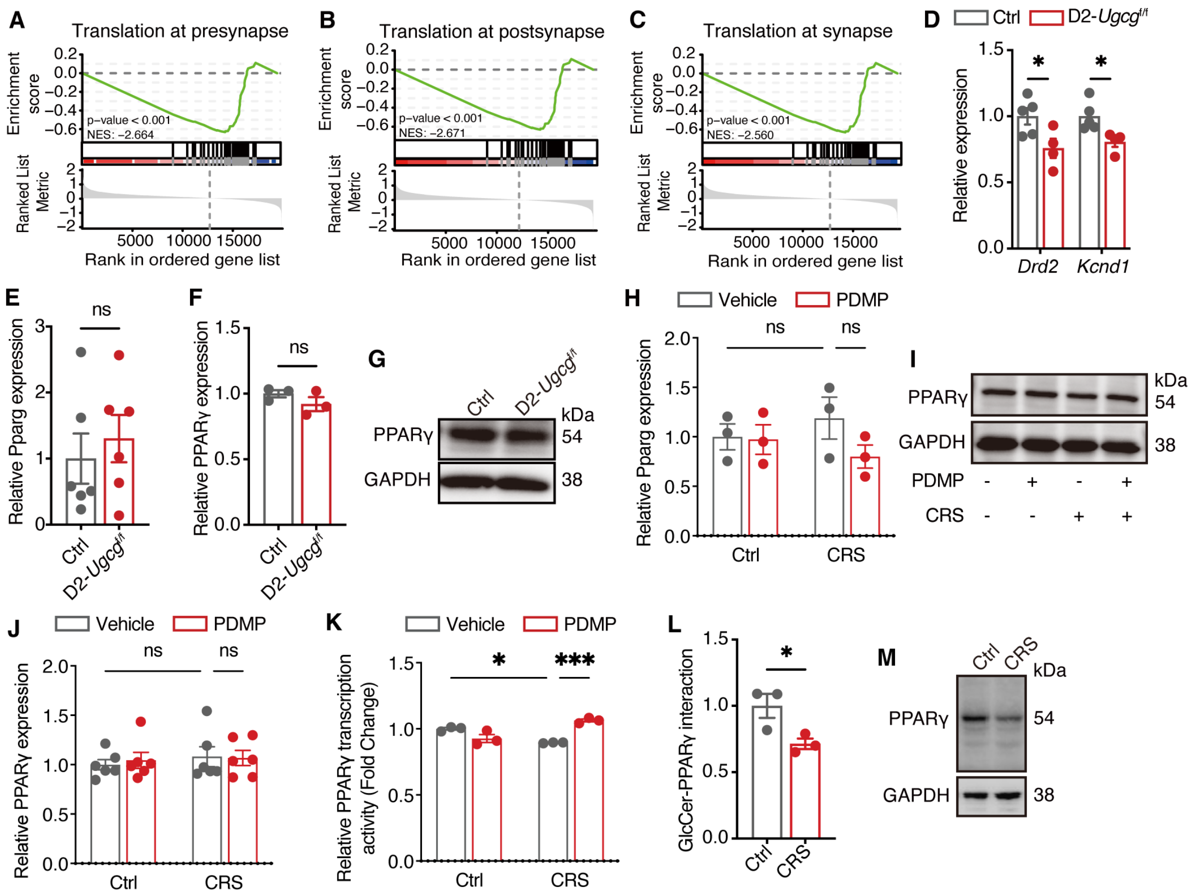

Figure S6. GCS modulates D2-MSNs PPARγ signaling in the dorsal striatum. (**A-C**) Gene set enrichment analysis (GSEA) shows significant enrichment of genes involved in translation at presynapse, postsynapse and synapse. (**D**) The mRNA levels of PPARγ target genes in the dorsal striatum of D2-*Ugcg*$^{f/f}$ and Ctrl mice. Ctrl, n = 5; D2-*Ugcg*$^{f/f}$, n = 4. **(E)** qRT-PCR analysis of *Pparg* (encoded PPARγ) in the dorsal striatum of D2-*Ugcg*$^{f/f}$ and Ctrl mice. n = 6. (**F**, **G**) PPARγ protein levels in the dorsal striatum of mice following *Ugcg* deletion. n = 3. **(H-K)** The *Pparg* mRNA (H), protein (I, J) and activity (K) in the dorsal striatum of CRS-induced mice following intra-dorsal striatum infusion of PDMP. **(L, M)** Lipid-protein pull-down assay shows PPARγ-GlcCer binding in the dorsal striatum of mice after CRS. n = 3. All data are presented as the mean ± SEM. Statistical significance was determined using two-sided unpaired t-test (D-F, L) or one-way ANOVA with Tukey's multiple comparisons test (H, J, K). *$p < 0.05$; ns, not significant.

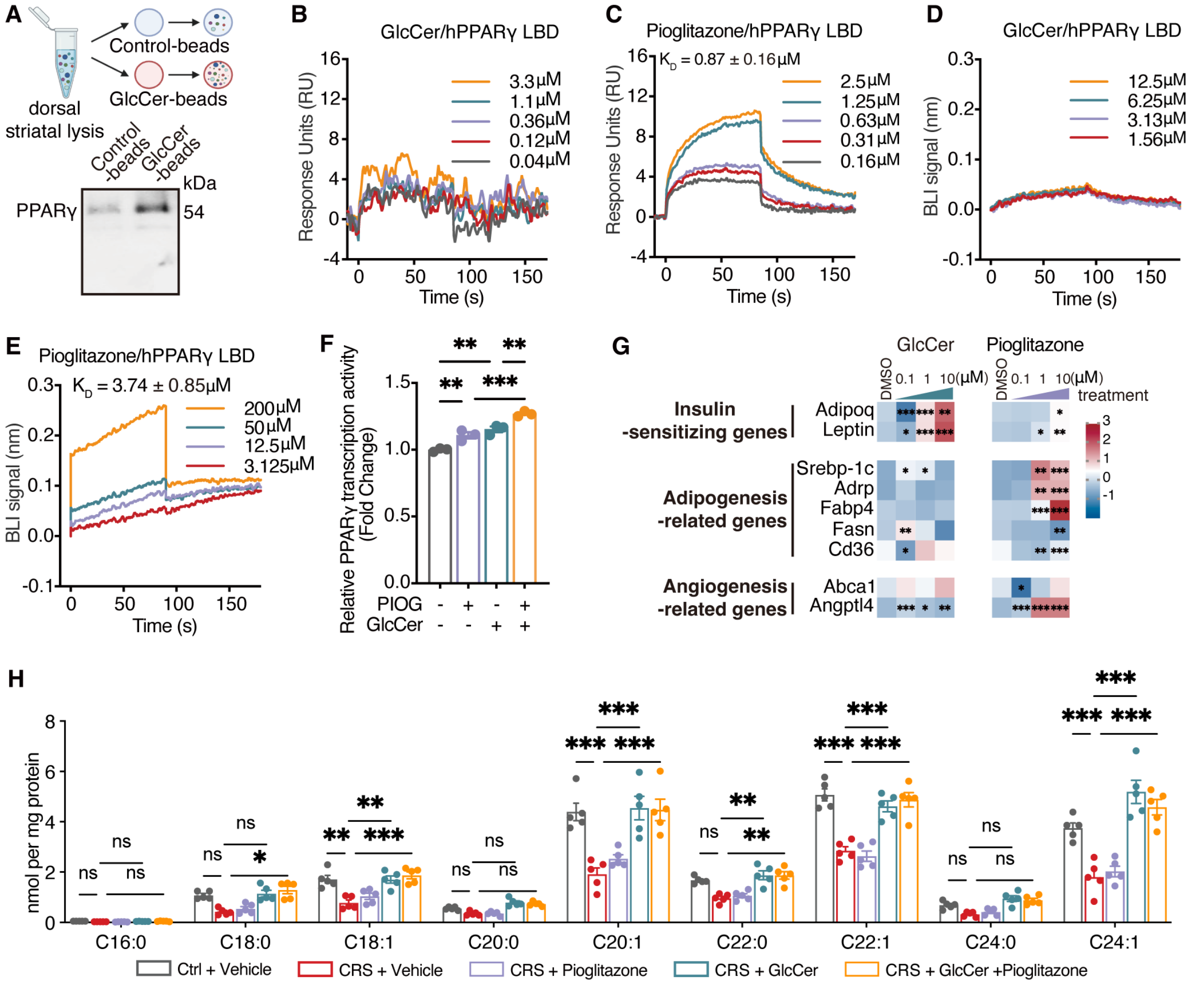

Figure S7. GlcCer regulates the expression of PPARγ target genes independent of binding with the LBD of PPARγ. (**A**) Schematic of the lipid-protein pull-down assay and representative images of GlcCer-hPPARγ binding in the dorsal striatum of mice. (**B, C**) SPR-based determination of K_D for hPPARγ LBD binding to GlcCer (B) and pioglitazone (C). RU, response units. (**D**, **E**) The binding affinity between hPPARγ LBD and GlcCer was measured by BLI (D), and pioglitazone was used as a positive control (E). **(F)** PPARγ transcriptional activity in N2a cells treated with GlcCer, pioglitazone or their combination. n = 3. (**G**) Exposure to GlcCer (0.1, 1 and 10 μM) or pioglitazone (0.1, 1 and 10 μM) altered the expression of PPARγ target genes in N2a cells. n = 3. **(H)** The level of GlcCer with different carbon chain lengths in the dorsal striatum of CRS-induced mice following intra-dorsal striatum infusion of pioglitazone, GlcCer, or their combination. n = 5. All data are presented as the mean ± SEM. Statistical significance was determined by one-way ANOVA with Tukey's multiple comparisons test (F, G, H). *$p < 0.05$; **$p < 0.01$; ***$p < 0.001$.

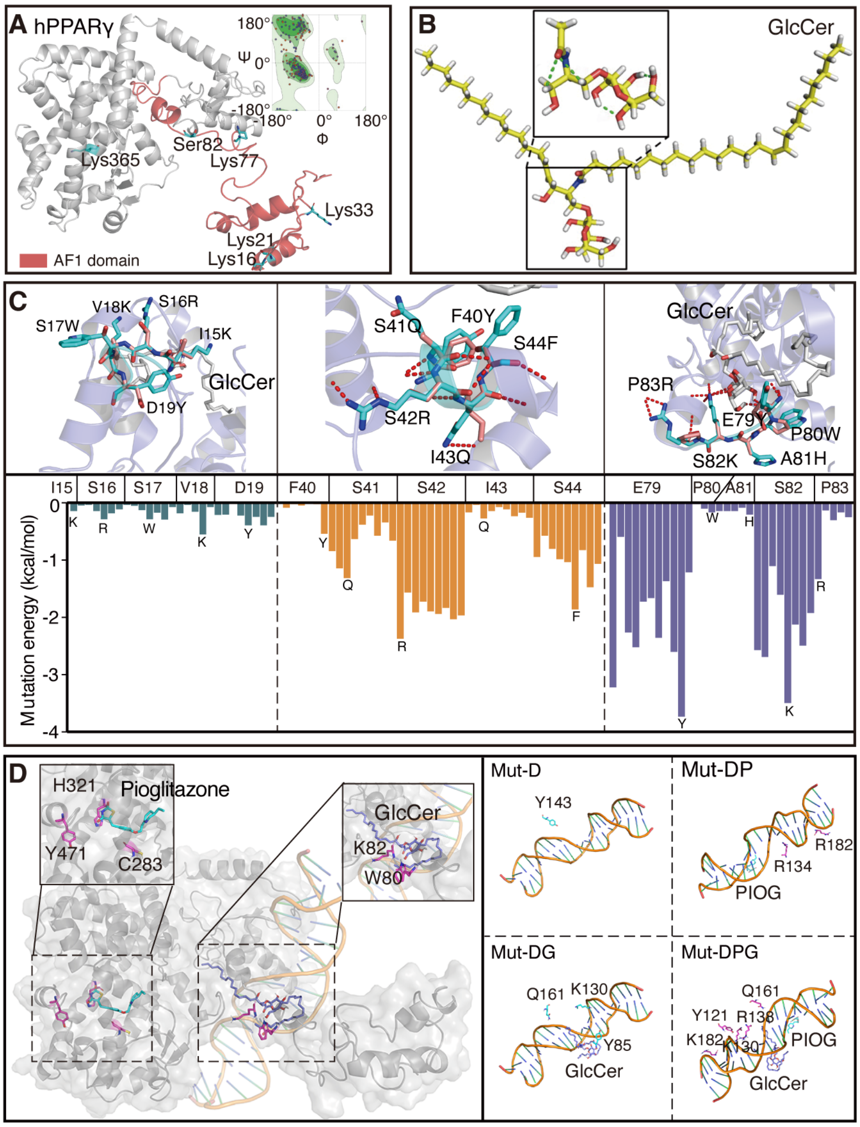

Figure S8. Molecular dynamics simulation between GlcCer and hPPARγ. (**A**) Cartoon representation of full-length hPPARγ structure. (**B**) Chemical structure of GlcCer. (**C**) Molecular docking analysis shows GlcCer interaction with three hPPARγ mutants, including Δ15-19, Δ40-44 and Δ79-83 (Top) and their mutation energies (Bottom). (**D**) Molecular dynamics simulations illustrate the interaction of the hPPARγ Δ79-83 with DNA, GlcCer and pioglitazone (Left), and a detailed representation of hydrogen bonds in Mut-D, Mut-DP, Mut-DG and Mut-DPG (Right). Mut-D: hPPARγ Δ79-83 bound to DNA; Mut-DP: Mut-D with pioglitazone; Mut-DG: Mut-D with GlcCer; Mut-DPG: Mut-D, pioglitazone and GlcCer.

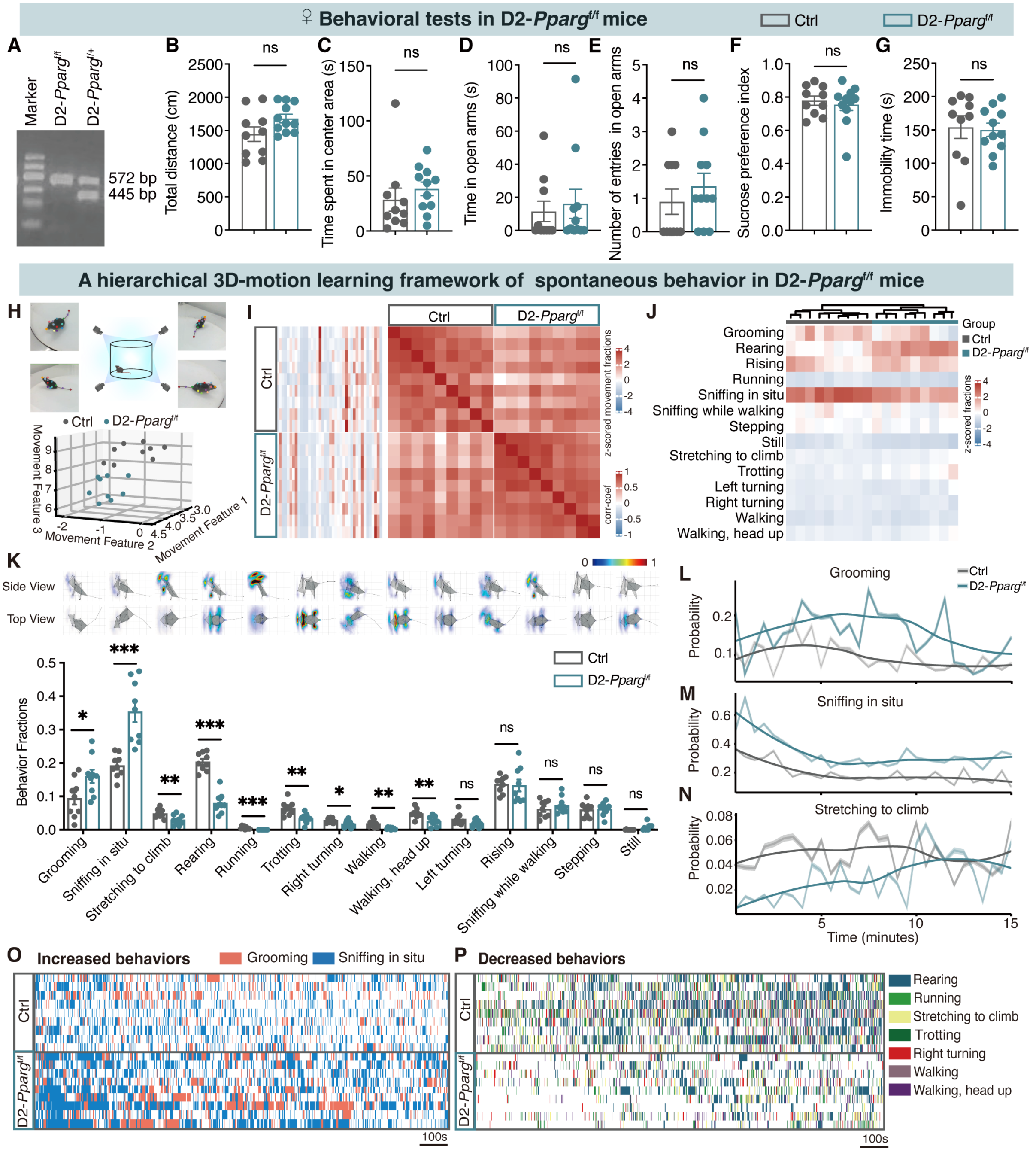

Figure S9. Deletion of *Pparg* in D2-MSNs impairs spontaneous behavior profiles and decreases calcium signaling in the dorsal striatum. (**A**) Representative PCR genotyping of D2-MSNs-specific *Pparg* heterozygous knockout (*Pparg*$^{f/+}$) and homozygous knockout (*Pparg*$^{f/f}$) mice. **(B-G)** Statistical analysis of the Ctrl and D2-*Pparg*$^{f/f}$ female mice in the OFT (B, C), EPMT (D, E), SPT (F), and FST (G). n = 10. **(H)** Top: workflow of mouse behavior recording and analysis using a 3D-motion learning framework. Bottom: Low-dimensional representation of the two animal groups, with gray dots represent samples from Ctrl group and cyan dots represent samples from D2-*Pparg*$^{f/f}$ group. n = 9. (**I**) Clustergram shows samples with 40 spontaneous behaviour modules. (**J**) Heatmap of 14 behavior movements in Ctrl and D2-*Pparg*$^{f/f}$ mice. (**K**) Top: Average skeletal representation of 14 behavior movements. Bottom:

Comparison of the fractions of different movement types observed in Ctrl and D2-*Pparg*$^{f/f}$ mice. *n* = 9. (**L-N**) Probability of behavior movements across time. (**O**, **P**) Ethograms of the two significantly increased movements (O) and seven significantly decreased movements (P) in mice. All data are presented as the mean ± SEM. Two-sided unpaired t-test was used (B-G, K). *p < 0.05; **p < 0.01; ***p < 0.001; ns, not significant.

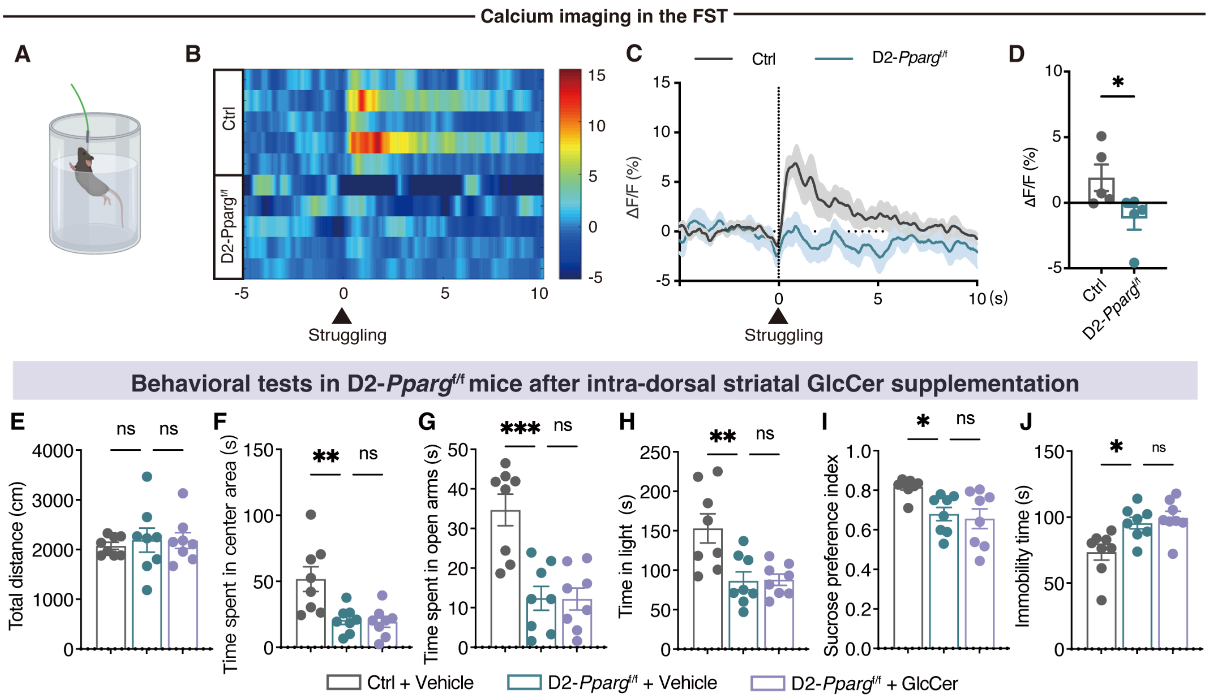

Figure S10. Dorsal striatal GlcCer-PPARγ signaling modulates neuronal calcium dynamics and depressive-like behaviors. (**A**) Schematic illustration of fiber placement for calcium imaging during the FST. (**B**) Representative heatmaps of GCamp6m transient ΔF/F events locked to struggling behavior, with each row representing one animal. n = 5. (**C**, **D**) Average and peak ΔF/F changes during struggling in the FST. n = 5. **(E-J)** Statistical analysis of D2-*Pparg*$^{f/f}$ and Ctrl mice after GlcCer microinfusion in OFT (E, F), EPMT (G), LDT (H), SPT (I), FST (J). n = 8. Data are presented as the mean ± SEM. Statistical significance was assessed using two-sided unpaired t-test (D) or one-way ANOVA with Tukey's multiple comparisons test (E-J). *$p < 0.05$; ns, not significant.

Table S1. The primers for qRT-PCR analysis.

Target	Sequence (5' → 3')
Fabp4	F: AACACCGAGATTTCCTTCAAACTGG<br>R: GTTATGATGCTCTTCACCTTCCTGTC
Leptin	F: GTTCCTGTGGCTTTGGTCCTATC<br>R: CCTGGTGACAATGGTCTTGATGAG
Angptl4	F: CCAGCAGCAGAGATACCTATCAAAG<br>R: CGAAGTCTTGTCTACTCCATTGTCTAG
Srebp-1c	F: AAGGATGCCCGTGCCAGTG<br>R: CCAGGTTAGAAGCAGCAAGATGTC
Abca1	F: TGGTGTTCTTCCTCATTACTGTTCTG<br>R: CCGCCTCACATCCTCATCCTC
Adipoq	F: GCCGCTTATGTGTATCGCTCAG<br>R: CCGTCATAATGATTCTGTTGGTTGTAG
Adrp	F: TGGCAGCAGCAGTAGTGGATC<br>R: CTGACATAAGCGGAGGACACAAG
Cd36	F: GCAGGTCTATCTACGCTGTGTTCG<br>R: TGTCTGGATTCTGGAGGGGTGATG
Fasn	F: TGCCCGAGTCAGAGAACCTACAG<br>R: TCCATAGAGCCCAGCCTTCCATC
Pparg	F: CCAAGAATACCAAAGTGCGATC<br>R: TCACAAGCATGAACTCCATAGT
Adora2a	F: CATCAACTGCTTCACCTTCTTC<br>R: GATCCTGTAGGCGTAGATGAAG
Kcnd1	F: TCCGTTTGGCAAAGAGTGGT<br>R: AGCTCGTCTGTGAACTCGTG
Ugcg	F: GAGAGACACCAGGGAGCTTG<br>R: GTGGCTGATGCATTTCATGT
Gapdh	F: AGGTCGGTGTGAACGGATTTG<br>R: TGTAGACCATGTAGTTGAGGTCA

Table S2. Hydrogen bonds between DNA and hPPARγ Δ79-83.

System	H-bonds	H-bonds between DNA and hPPARγ Δ79-83
Mut-D	47	DT4009-Tyr143
Mut-DP	62	DA3003-Arg134, DT4018-Arg182
Mut-DG	50	DC4013-Tyr85, DT4009-Gln161, DA3007-Lys130
Mut-DPG	70	DG3008-Tyr121, DA3007-Tyr121, DT4019-Arg182, DG3009-Arg138, DA3007-Lys130, DT4009-Gln161

Table S3. Binding free energy between DNA and hPPARγ Δ79-83 (kcal/mol).

Systems	ELE_{IN}[a]	VDW_{IN}[b]	ELE_{GB}[c]	VDW_{GB}[d]	H[e]	TS[f]	ΔG
Mut_D	-4135.70±46.94	656.64±2.62	4173.13±25.19	-12.72±0.05	681.35±53.34	691.48±35.91	-10.13
Mut_DP	-4017.10±19.25	655.16±6.17	4062.33±31.59	-13.00±0.14	687.38±37.50	702.94±31.82	-15.56
Mut_DG	-4022.55±34.00	643.09±2.60	4072.29±31.31	-13.03±0.12	679.80±46.29	690.85±15.78	-11.05
Mut_DPG	-3973.29±4.22	652.46±0.67	4022.68±30.64	-12.87±0.02	688.89±30.94	706.87±30.26	-17.98

[a]ELE_{IN} refers to the electrostatic binding energy in vacuum; [b]VDW_{IN} refers to the van der Waals binding energy in vacuum; [c]ELE_{GB} and [d]VDW_{GB} refer to the polar and non-polar parts of the solvation effect, respectively; H[e] represents the enthalpy change, which equals the sum of ELE_{IN}, VDW_{IN}, ELE_{GB} and VDW_{GB}.
